# The global biological microplastic particle sink

**DOI:** 10.1038/s41598-020-72898-4

**Published:** 2020-10-07

**Authors:** K. Kvale, A. E. F. Prowe, C.-T. Chien, A. Landolfi, A. Oschlies

**Affiliations:** 1 GEOMAR Helmholtz Centre for Ocean Research, West Shore Campus, Duesternbrooker Way 20, 24105 Kiel, Germany; 2 ISMAR-CNR, Via Fosso del Cavaliere, 100, 00133 Rome, Italy

**Keywords:** Biogeochemistry, Climate sciences, Ocean sciences

## Abstract

Every year, about four percent of the plastic waste generated worldwide ends up in the ocean. What happens to the plastic there is poorly understood, though a growing body of evidence suggests it is rapidly spreading throughout the global ocean. The mechanisms of this spread are straightforward for buoyant larger plastics that can be accurately modelled using Lagrangian particle models. But the fate of the smallest size fractions (the microplastics) are less straightforward, in part because they can aggregate in sinking marine snow and faecal pellets. This biologically-mediated pathway is suspected to be a primary surface microplastic removal mechanism, but exactly how it might work in the real ocean is unknown. We search the parameter space of a new microplastic model embedded in an earth system model to show that biological uptake can significantly shape global microplastic inventory and distributions and even account for the budgetary “missing” fraction of surface microplastic, despite being an inefficient removal mechanism. While a lack of observational data hampers our ability to choose a set of “best” model parameters, our effort represents a first tool for quantitatively assessing hypotheses for microplastic interaction with ocean biology at the global scale.

## Introduction

How much and where the ocean is accumulating plastic is an open question. Lagrangian modelling of wind-driven particles simulate rapid accumulation at the surface of ocean gyres^1–6^ and the North Atlantic^2^. These findings do not disagree with the sparse observational record^1–3,6–13^, which is biased towards these same locations. Large, floating plastic detritus has historically captured the largest share of attention by the scientific community, the media, and the public. However, budgetary analysis of the surface layer has revealed a substantial “missing” fraction of plastic, that is, plastic that is expected to be present in the surface but is not found with neuston nets^1,3^. The majority of this missing fraction is in the smaller plastic particle size classes, i.e., the microplastic (MP; 0.1 to 5 mm in diameter) and smaller fractions^1,3^. These small plastic size fractions are both directly introduced to the ocean from outside via rivers, wind, and boats, and form within the ocean during the physical and photochemical breakdown of larger plastic items. Recent observations reveal high concentrations of MP outside of the well-known accumulation zones, such as the deep ocean^14–16^, East Asian seas around Japan^13^, and Arctic^2,17^.

Tiny plastic particles missing from the surface ocean have several possible removal pathways. Some types of plastic are negatively buoyant, and start to sink to the sediments directly from entry into the water column. The transport of negatively buoyant plastic particles is probably dominated by seafloor currents^18^. For positively buoyant plastic particles there exist two removal pathways. The first is abiotic removal, where the plastics lose their buoyancy during physical or photochemical breakdown into smaller size classes and become neutrally buoyant, advecting as a passive tracer. The second possibility is biological uptake and removal. Zooplankton have been observed consuming MP particles and egesting them in pellets^19,20^, providing potentially rapid export out of the surface layer. Marine snow aggregates have also been observed to aggregate with MP^21–23^, which offers a similar pathway for surface plastic removal. Fish or other animals might also be accumulating a significant stock of microplastic in their biomass, though the authors could find no global estimate of their potential contribution to the “missing” fraction. Non-removal possibilities also exist, such that microplastics remain at the ocean surface but are simply too small to be captured and counted by neuston nets^24^, or that microplastic inputs are over-estimated. In any case, wind-driven surface modelling might not be sufficient for simulating MP transport and predicting accumulation zones.

Physical breakdown was modelled in a global budgetary fashion^25^, and biological transport was approximated using spatially constant biological removal rates^6^ and prescribed subsurface particle release^26^. Accounting for subsurface transport in a Lagrangian model changes MP distributions in the ocean, moving a significant fraction to the high latitude sub-surface^26^. But it does not simulate the surprisingly high accumulation rates reported in trenches^15^, where concentrations of MP are reported to exceed 2000 particles per cubic meter, roughly 500 times greater than in the surface waters around Japan^13^. Eulerian ocean modelling was recently applied to plastics transport to examine the effects of buoyancy and idealized removal on plastics accumulation^18^. They predicted potentially large quantities accumulating in the deep sea (negatively buoyant particles) and throughout the water column (neutrally buoyant particles). That both particle buoyancy and subsurface particle release^26^ can strongly affect the development of accumulation zones suggests that biological transport might be similarly influential in shaping global plastic distributions. In this case, consideration of the spatial co-occurrence of biologically-active plastic and biology would be important in predicting plastic accumulation zones.

Exactly how important biologically mediated plastic transport might be relative to purely physical transport is unquantified at a global scale. It is widely assumed in the literature that biology is taking up and exporting a significant portion of the global surface MP, but a simple budgetary analysis^27^ using a biological particle flux estimate derived from an earth system model reveals a major mismatch between the amount of plastic the ocean biology can take up (given a certain set of conservative assumptions) and what it is apparently removing. There is so much marine snow production in the ocean, even in the gyres, that the standing stock of MP ought to be removed throughout most of the surface ocean within less than 2 years. That the latest survey results suggest MP is continuing to increase at the surface^28^, despite large potential biological removal rates^27^, suggests the intriguing possibility that a widespread entrainment/release cycle dominates the transport of biologically-active plastic particles. In this scenario, aggregation of MP into marine snow, consumption of MP by zooplankton, and colonization of MP by marine algae (biofouling^29^), all produce plastic-organic aggregates that become negatively buoyant and sink to the depth the organic material is remineralized. Upon remineralization the particles are released from their organic matrix, potentially rising back to the euphotic zone. If biologically-mediated plastic transport is indeed a major pathway for the biologically-active plastic fraction in the ocean, it is expected that the resulting accumulation patterns would differ from purely positively, neutrally, or negatively buoyant particles.

We explore the biological MP sink using a model that takes a novel approach to the simulation of MP by using an Eulerian representation of MP embedded in an earth system model, in which plastic released to the ocean from coastlines and major shipping lanes is explicitly taken up and released by marine snow and eaten by zooplankton. We do this to see if we can simulate the observed missing surface fraction^1,3^, and to see if missing surface MP can be reconciled with hypothesised inefficient biological removal^27^. Although our model does not simulate size classes we refer to it as MP, because the plastic represents the plastic fraction that can be considered biologically active for marine algae and small zooplankton, as are typically applied to biogeochemical ocean models to represent the base of the marine food web. The main text focuses on a subset of the full envelope of parameter uncertainty detailed in the Supplementary Information. In the Supplementary Information, we test the sensitivity of MP distribution to physical transport, marine snow and faecal pellet uptake parameters and then reduce the sample size based on criteria described below.

Explicit modelling of biological interactions with MP in an earth system model offers another advantage: a predictive element of how these interactions could change in the future, as climate change alters the ocean circulation and ecosystem. We extrapolate historical trends in global MP pollution rate to the year 2100, in combination with rapid increases in atmospheric $\hbox{CO}_2$, to examine how the ocean biological sink of MP might change into the future.

## Results

The three simulations presented in the main text (Table 1) were selected to represent the range of “free” (unattached) MP surface concentrations produced within a 14-member subset of the third 300-member Hypercube (see Experimental Setup and Supplemental Information). This subset was produced by removing all samples of the Hypercube that were numerically unstable or using plastic input rates that greatly exceed the estimated annual global plastic release to the ocean^30^ and marine snow particle aggregation rates far outside the estimated average range measured in the open ocean^31^.

**Table 1:** Parameter values for the sample simulations analysed here.

Sim.	$F_T$	$F_R$	$F_B$	$F_{A}$	$k_P$	$\psi _{MP}$	$R_{F:MP}$
No Bio	0.260	0.033	–	–	–	–	–
TestLo	0.137	0.003	0.132	0.003	0.424	0.260	1.029
TestHi	0.329	0.074	0.888	0.098	833.290	0.132	1.489
TestMed	0.276	0.011	0.528	0.092	615.508	0.193	0.993

Please see the “Methods” section for parameter definitions.

All members of the 14-simulation subset contain total MP particle inventories that roughly agree with an independently calculated MP inventory at year 2010^3^. Four of the simulations also produce power law-shaped “free” MP particle profiles that generally agree with new observations from the North Pacific Gyre^32^. Local surface or sub-surface particle minima in the unattached MP compartment are also present in 12 of the simulations, which is broadly consistent with the findings of widespread “missing” microplastic in surface sampling^1,3^. Our simulations furthermore simulate observed local sub-surface particle maxima^16,32^ in 9 samples, which are produced as marine snow-aggregated microplastic particles ($\hbox{MP}_A$) and zooplankton faecal pellet-bound plastic particles ($\hbox{MP}_Z$) release MP back into the free MP compartment. However, note the data compilation^32^ included sizes of MP within the larger end of the size range than are typically thought to aggregate with marine snow^33^ or that are ingested by small zooplankton^34^. Comparison to data obtained from manta trawls, such as used by^32^ biases the comparison away from what might be the most biologically-active size range (although, our model does not simulate size classes). Nevertheless, it is interesting that biological uptake can apparently reproduce characteristics of the observed profiles.

In the main text, TestLo represents low surface MP concentrations and efficient marine snow uptake of MP, while TestHi represents a high MP particle load and large surface MP concentrations. TestMed is the moderate configuration, with a moderate MP inventory and moderate surface concentrations. The suite of three simulations represent a spread in the fractional buoyancy of the unattached MP partition, in the amount returning to the water column from the seafloor, and in the zooplankton grazing preference for MP. The food:MP substitution ratio $R_{F:MP}$ is greater than 1 in 2 out of 3 simulations, which means more than 1 g of food is replaced by 1 gram of MP in a zooplankton’s diet. The sparsity of water column profile data on MP concentrations available from the real ocean makes it difficult to conclude any simulation is more or less realistic than another, although the new depth profiles from the North Pacific Gyre^32^ share a common power law shape as TestHi. Note also that all simulations presented include both increasing atmospheric $\hbox{CO}_2$ concentration forcing and increasing rates of plastic pollution and cannot be considered steady-state. We compare these three simulations of biological MP uptake to one without, selected from the sample space of the no-biological-uptake simulations based on its physical MP transport parameter values being closest to the average of the three biological transport simulations (referred to here as No Bio; Test99 in the supplied dataset).

### Biology offers microplastic a path to the deep sea

**Figure 1:**
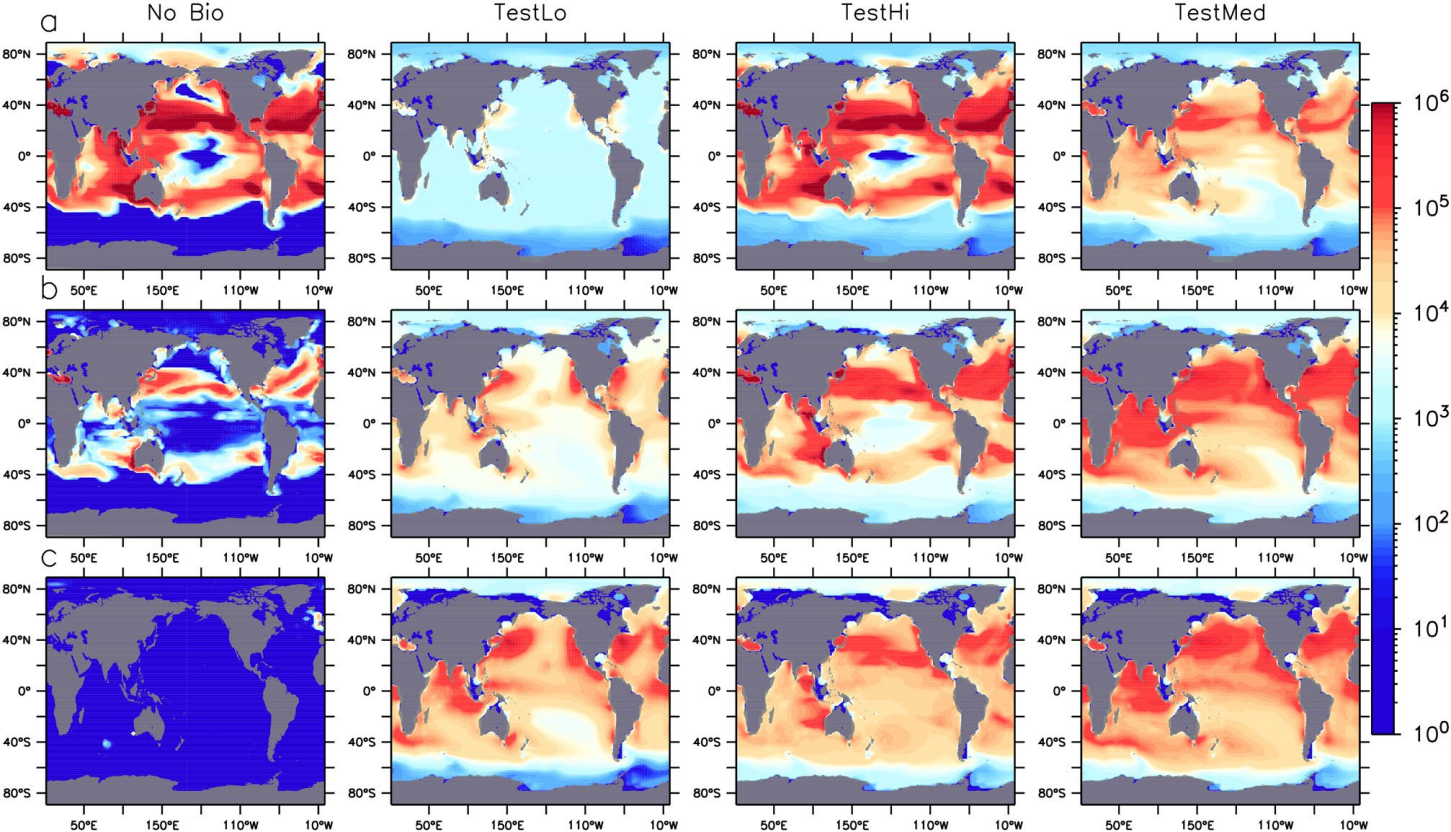
Total MP water column particle inventories (all three MP partitions) in particles km$^{-2}$ in three depth brackets (0:100 m; **a** row, 100:500 m, **b** row, and below 500 m, **c** row), at year 2020 in four models, one with no biological uptake (left) and three in which model parameters differ (see Table 1). Figure was generated using the Ferret plotting program version 6.82. Ferret is a product of NOAA’s Pacific Marine Environmental Laboratory http://ferret.pmel.noaa.gov/Ferret/.

All simulations of biological transport remove MP from the upper 100 m of the ocean, moving particles to the sub-surface (100–500 m depth) and below (Fig. 1). Biological transport can reduce the amount of MP found in gyres and diffuse the inventory moving poleward with western boundary currents, spreading it into the intermediate and deep North Pacific and Atlantic basins and into the southern Indian Ocean via the Aghulas Current. TestLo has the lowest plastic pollution rate ($F_T$ is 13.7% of total annual waste generation), as well as the most efficient marine snow aggregate MP removal coefficient ($k_P=0.424$ particles $\hbox{m}^{-3}$) and the smallest fraction that returns to the water column at the seafloor ($F_B=13.2$%), which together account for its relatively lower overall particle inventory and smallest surface concentrations, relative to the other two models. TestLo also has the lowest fraction of MP assigned a rise rate ($F_R=0.3\%$), which also reduces surface MP concentrations. TestHi has the highest plastic pollution and seafloor return rates ($F_T=32.9$% and $F_B=88.8$%, respectively), which accounts for its relatively greater particle load than the other two models. Potential sub-surface accumulation hotspots include the coastal zones of east Asia as well as the east and west coasts of the US and Canada, Spain and Portugal, the Gulf of Guinea and the Tasman Sea between Melbourne and Sydney. The highly biologically productive southeast Asian seas and northern Indian Ocean are also demonstrated to be potentially significant accumulation regions, with biologically-aggregated MP spreading across the Indian Ocean basin and following both the Indonesian Throughflow (to the west) and South Equatorial Currents (to the east). Deep ocean accumulation is demonstrated in these same regions, as well as off the west coast of Latin America and in the Eastern Boundary Upwelling Systems. These results differ from modelling of negatively and neutrally buoyant plastics^18^, in particular in our simulations’ representation of major accumulation in the deep North Atlantic and Pacific basins, as well as in significant subsurface Equatorial trapping. These differences might be due to both the mechanism of transport, as well as differences in the spatial arrangement and magnitude of our respective MP release assumptions.

**Figure 2:**
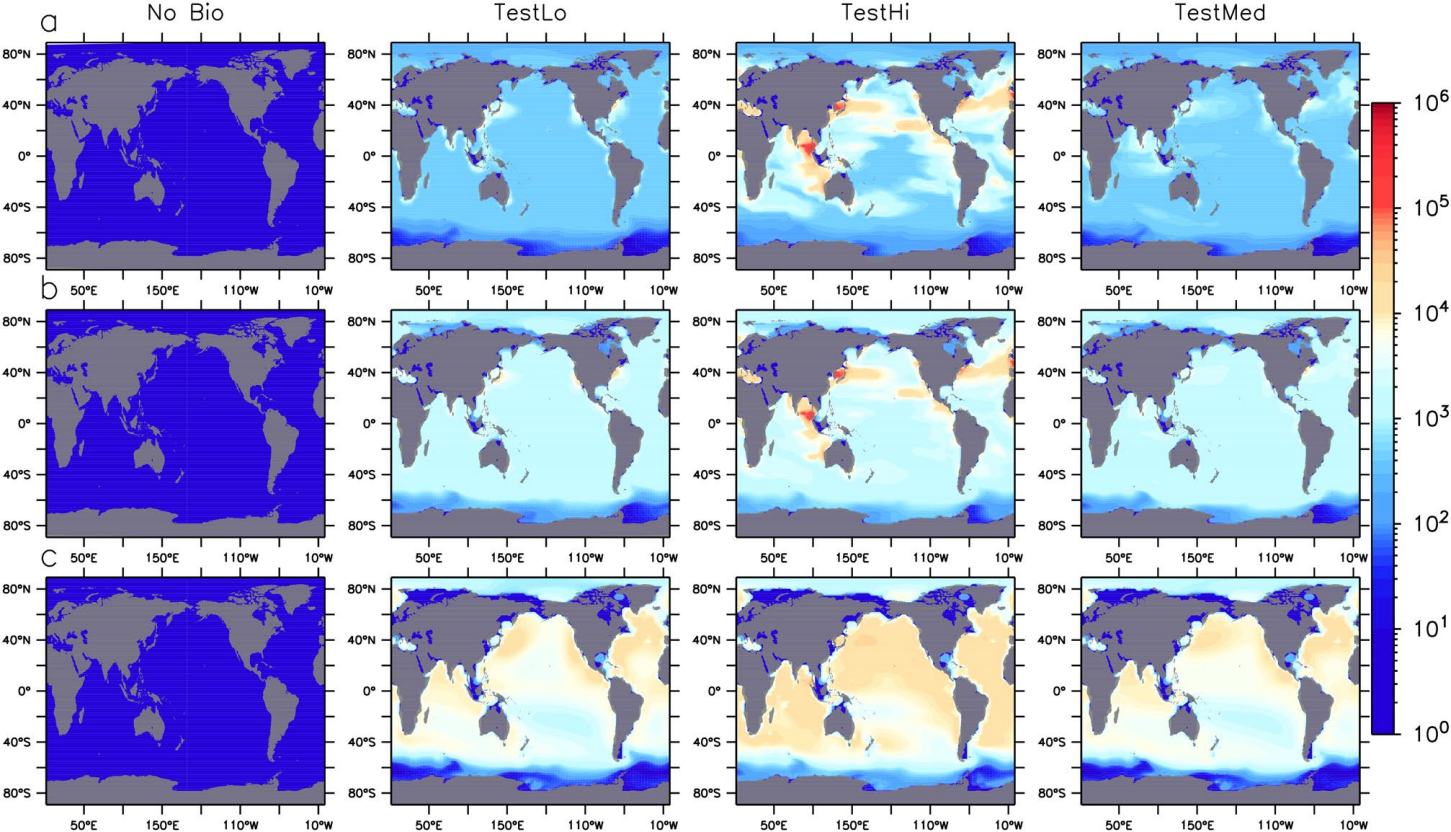
Marine snow-associated MP water column particle inventories in particles km$^{-2}$ in three depth brackets (0:100 m, **a** row, 100:500 m, **b** row, and below 500 m, **c** row), at year 2020 in four models, one with no biological uptake (left) and three in which model parameters differ (see Table 1). Figure was generated using the Ferret plotting program version 6.82. Ferret is a product of NOAA’s Pacific Marine Environmental Laboratory http://ferret.pmel.noaa.gov/Ferret/.

**Figure 3:**
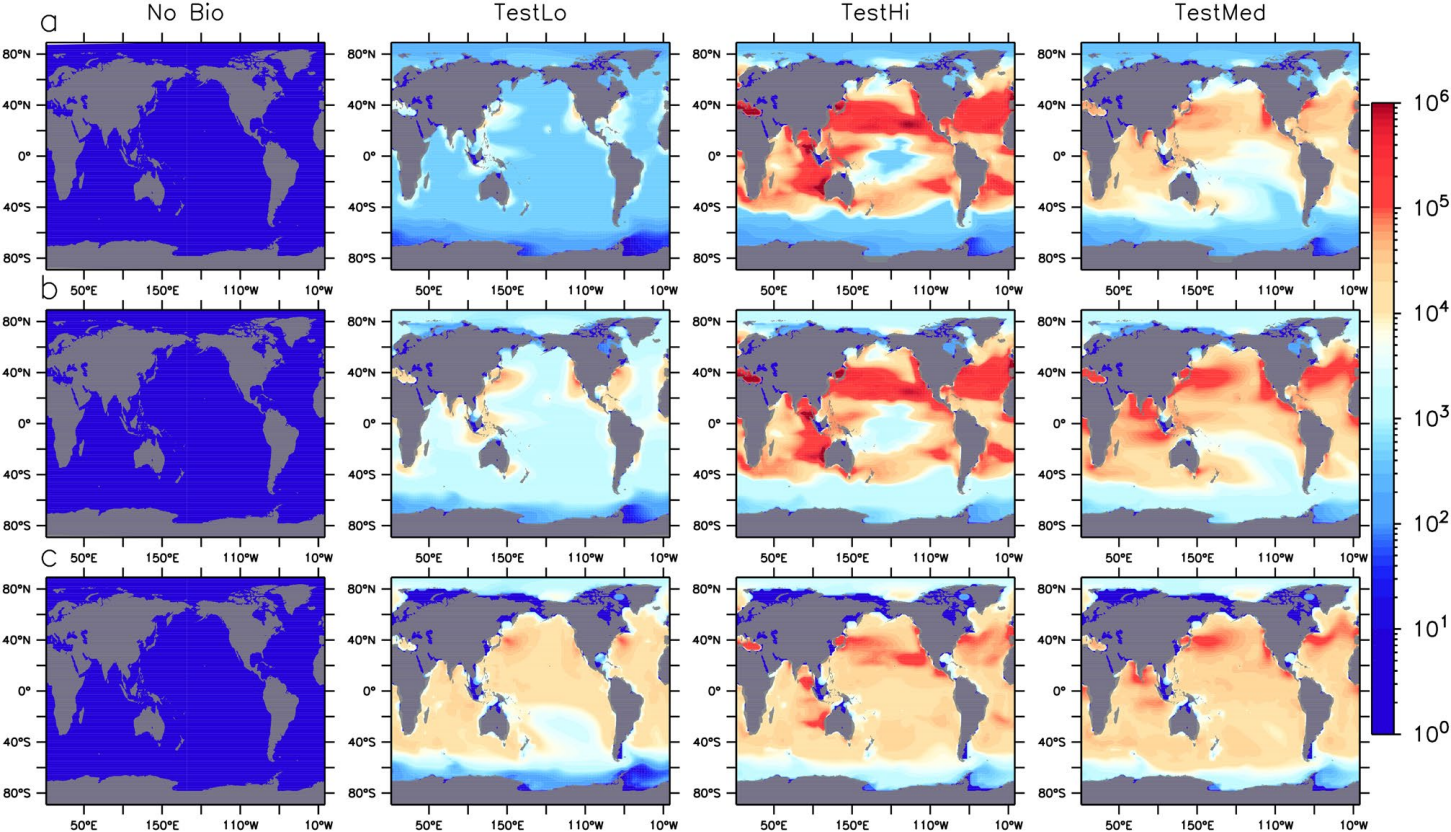
Zooplankton pellet-associated MP water column particle inventories in particles km$^{-2}$ in three depth brackets (0:100 m, **a** row, 100:500 m, **b** row, and below 500 m, **c** row), at year 2020 in four models, one with no biological uptake (left) and three in which model parameters differ (see Table 1). Figure was generated using the Ferret plotting program version 6.82. Ferret is a product of NOAA’s Pacific Marine Environmental Laboratory http://ferret.pmel.noaa.gov/Ferret/.

Marine snow aggregation is the weaker biological pathway for MP compared to aggregation in faecal pellets in all simulations presented here (compare $\hbox{MP}_A$ held in aggregates, Fig. 2 with $\hbox{MP}_Z$ held in zooplankton pellets, Fig. 3). The geographical distribution is similar to, but not exactly the same as for the total MP distribution. Most notably, MP held in aggregates does not show high concentrations in the gyres; the greatest concentrations are found in and downstream of the most biologically-productive regions that also contain significant MP concentrations (western boundary currents, upwelling regions). The distribution is also far more diffuse, particularly in the deeper ocean levels.

Zooplankton consumption and subsequent egestion of MP in pellets is the dominant biological pathway for MP transport in our model in all simulations presented here (Fig. 3), as well as in all 14 members of the Hypercube that met our selection criteria (Supplemental Fig. S9). Concentrations held in this partition are of the same order of magnitude as those held within the unattached MP partition (Supplemental Fig. S9). MP held in pellets also has a unique geographical distribution somewhat between that of the unattached MP (in that it is found in high concentrations in the gyres) and MP in marine snow (more diffuse patterns in the deep ocean). That pellet-bound MP is simulated to accumulate in the gyres suggests the substitution of MP for food in these low-productivity regions is significant.

### How efficient are the biological microplastic sinks?

The above analysis demonstrates a potentially influential role for biology in shaping MP distributions in the global ocean, transporting it both into the deep ocean and across major ocean basins. How can this result be reconciled with previous offline calculations that hypothesized biology as an inefficient surface removal mechanism^27^?

**Figure 4:**
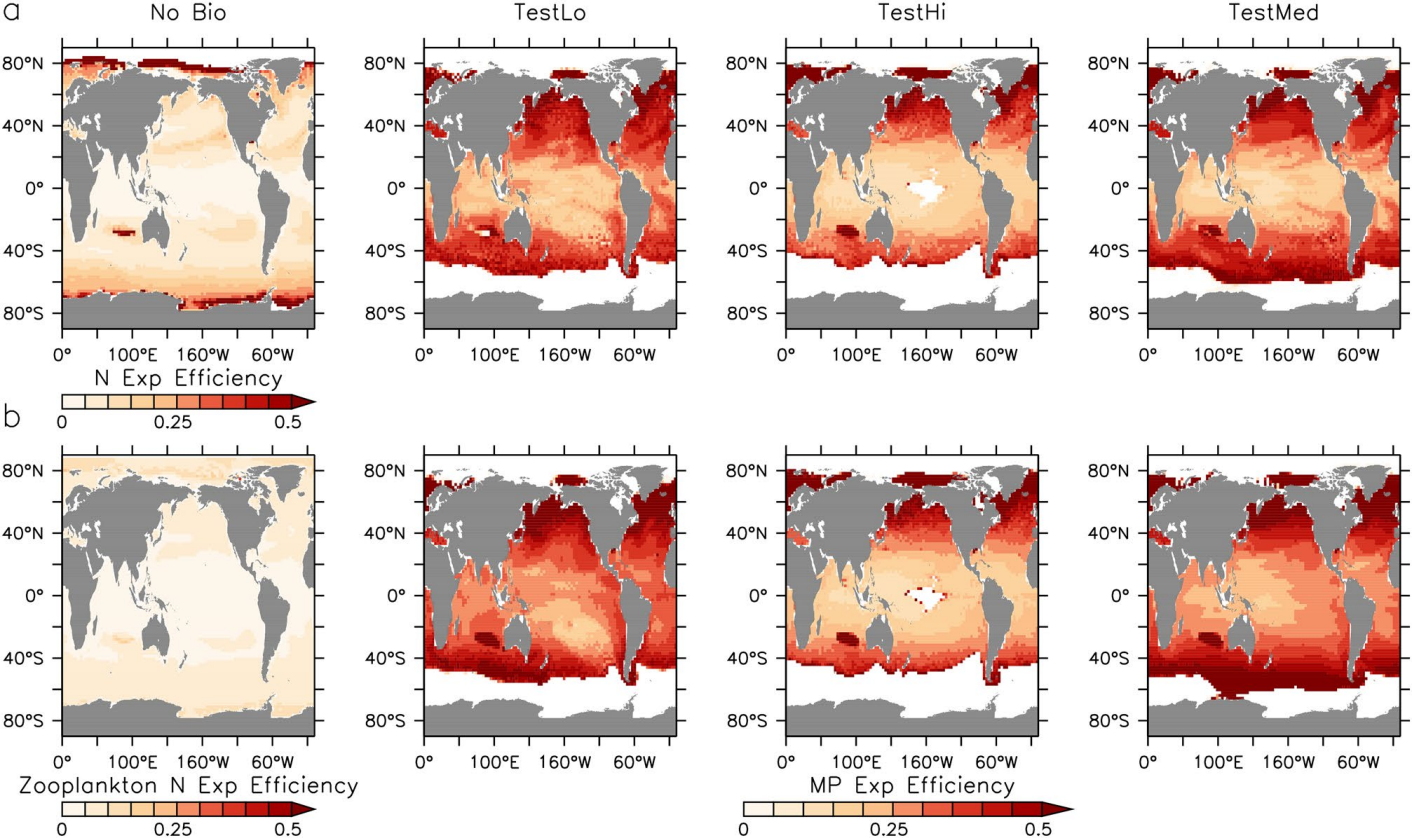
Nitrogen and MP export efficiency (as defined in text) for marine snow (**a**) and zooplankton faecal pellets (**b**) at year 2020. MP export efficiency is zero in the high latitudes due to zero MP there. Figure was generated using the Ferret plotting program version 6.82. Ferret is a product of NOAA’s Pacific Marine Environmental Laboratory http://ferret.pmel.noaa.gov/Ferret/.

The possibility of a widespread entrainment/release cycle occurring in the upper ocean^27,35^ is assessed in our simulations by defining MP export efficiencies for both marine snow and faecal pellets (Fig. 4), as $\hbox{MP}_A$ (marine snow aggregated MP) and $\hbox{MP}_Z$ (zooplankton pellet bound MP) export rates at the second model depth layer (130 m) divided by their respective integrated marine snow and zooplankton MP uptake rates above that depth. We also plot particulate organic nitrogen (PON) export efficiency for both detritus types (calculated as detrital export at the second model depth divided by integrated net primary production; NPP, and grazing above that depth) for the purposes of comparison. Our model includes temperature dependencies for primary production, zooplankton grazing, and detrital remineralisation, and the effect of these on PON export efficiency can be seen in generally greater efficiency in cooler regions and in gyres, where the increase in the maximum growth rate of zooplankton with temperature is capped above 20 $^\circ$C^36,37^ to represent metabolic limitation of growth in very warm water. High PON export efficiency apparent in the Indian Ocean gyre is an artefact of the calculation (there is near-zero NPP in those cells but some particle advection into the gyre). Likewise, lower temperatures also inhibit the release of MP from marine snow aggregates, increasing $\hbox{MP}_A$ export efficiency. $\hbox{MP}_A$ export efficiency is zero in the high latitudes due to there being effectively zero MP uptake (annually averaged) by marine snow there. Spatial patterns and magnitudes of $\hbox{MP}_A$ export efficiency are similar in the three models described here because the MP marine snow release rate is a prescribed function of temperature, and the $\hbox{MP}_A$ sinking rate is a prescribed function of depth, both of which are the same across models. The magnitude of export efficiency for MP compared to PON is bigger because there are no losses to the microbial loop for MP held in aggregates.

Zooplankton PON export efficiency is lower than that of marine snow (Fig. 4) due to the assimilation of grazed N into zooplankton body mass and losses to metabolism. However, faecal pellets have a similar MP export efficiency as marine snow because the MP is not assimilated (a 100% excretion rate) and hence there are no losses except from pellet remineralisation. Relative to nutrients, transport of MP by biology via both pathways can be considered “efficient” in that by virtue of its inorganic nature there are no losses by respiration. However, over most of the global ocean, for every two MP particles taken up by biology in the surface, only 1 (or less than 1) will be exported out. In the tropics, only about 1 in 3 MP particles taken up will be exported. The MP particles left behind are then available to be taken up again, in a cycle of entrainment and release that can produce large surface and near-surface MP particle inventories despite widespread biological uptake.

### The future of the biological microplastic sink

**Figure 5:**
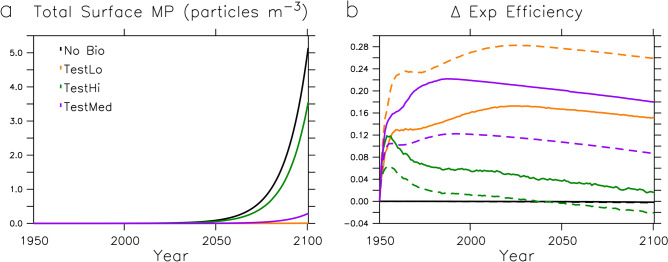
Surface grid cell average total MP concentrations (panel **a**) for each model configuration and change in nitrogen (black lines, panel **b**) and MP export efficiency (coloured lines, panel **b**) by marine snow (solid lines) and zooplankton faecal pellets (dashed lines).

MP export efficiency is expected to change in the future due to the coupled effects of environmental changes due to climate warming and increasing MP pollution rates. Figure 5 shows simulated changes in total MP surface concentrations, PON export efficiency, and marine snow and faecal pellet MP export efficiency over the timeseries. Strong marine snow uptake of MP in TestLo maintains average total surface concentrations near zero for the duration of the simulation, despite rapidly increasing MP pollution rates. However, less efficient biological removal in TestMed and TestHi allows surface concentrations to increase into the future.

By 2100, the ocean has become slightly less efficient in PON export due to warming, which raises remineralisation rates. Some regions in the middle and low latitudes have become more efficient; in our model the coccolithophore habitat expands due to surface stratification, and coccolithophores (using their shells as ballast) are more efficient exporters of PON. Regional gains and losses in PON export efficiency nearly compensate in the global mean (black lines in Fig. 5b). These physical and biological changes are identical across all model simulations. Global mean MP export efficiency of marine snow and zooplankton pellets follow similar trends (of different magnitudes) for each simulation (compare solid and dashed lines in Fig. 5b). MP export efficiency is simulated to have risen quickly with the onset of MP pollution in the 1950s and 1960s, but it is simulated to peak (prior to 1960 in TestHi, around 1990 in TestMed, and around 2030 in TestLo), and then decline. The decline in global MP export efficiency over the 2000s is driven by the reduction of PON export, but the magnitude is larger due to differences in the regional trends; MP concentrations remain higher in the low and middle latitudes where PON export largely decreases. Whereas increases in high latitude PON export compensate the low and middle latitude PON export declines in the global mean, significant amounts of MP never reach the high latitudes and the global trend is one of decline in all model configurations.

## Discussion

Here we present our new tool for quantitative assessment of microplastic interaction with ocean biology at a global scale. Large uncertainties in basic processes necessitated an extensive exploration of the parameter space, which is provided in the Supplemental Information. Our 700-member Latin Hypercube parameter ensemble produced 14 plausible parameter combinations that met a limited set of metrics. From these 14 simulations, we selected 3 to represent simulated low, medium, and high surface MP concentrations. The three simulations presented in the main text:

- are numerically stable and constrained to total MP inventories at or below the time-integrated global ocean pollution rate at year 2010
- are roughly constrained to an independently calculated plastic annual ocean pollution rate^30^
- are generously constrained to independently calculated marine snow aggregation rates^31^
- roughly agree with an independently calculated total ocean MP inventory for year 2010^3^
- produce subsurface MP particle maxima^16^ below local minima^32^
- quantitatively simulate the “missing” surface MP^3^.

The simulations presented here offer a first model exploration of explicit biological uptake and transport of passive and quasi-passive microplastic in the global ocean, over the historical past and into the future. We describe the mechanism of a widespread entrainment/release cycle that potentially strongly shapes MP inventory and distributions, and its vulnerability to ocean warming due to a changing climate. We show that even inefficient MP uptake parameters can lead to a transport of the MP inventory into deeper ocean levels, quantitatively simulating 100% of the observed “missing” surface microplastic. We find accumulation in the sub-surface radiating off coastlines along boundary currents and spreading into the deep North Atlantic and Pacific basins. We show that high levels of primary production, warm sea surface temperatures, and high plastic input rates can divert MP into the subsurface Eastern Boundary Upwelling Systems and along equatorial currents. We suggest that zooplankton faecal pellets may be the MP sink of greater magnitude relative to marine snow. However, large uncertainties still remain in the study of plastics and plankton and this model neglects an explicit representation of biofouling, modification of particle sinking rates^20–22,38^, higher trophic levels, plastic polymer type and plastic particle size. The particle size is relevant to the formation of marine aggregates, which mostly incorporate plastics smaller than 1 mm^33^, and to size-selection by zooplankton^34^. Zooplankton also demonstrate preferences for polymer type^34^, which we do not resolve. Abiotic degradation^39^ might reduce the exposure of biology to microplastic, and accounting for this explicitly might increase our biological uptake parameters. Updated information about historic and current MP pollution patterns from coastal regions and shipping lanes can be added to the model in the future. All of these factors are hypothesized to influence transport patterns and the resulting spatial distribution of simulated MP. We look forward to testing these various hypotheses through expanded capabilities in our model, as more data become available.

## Methods

**Figure 6:**
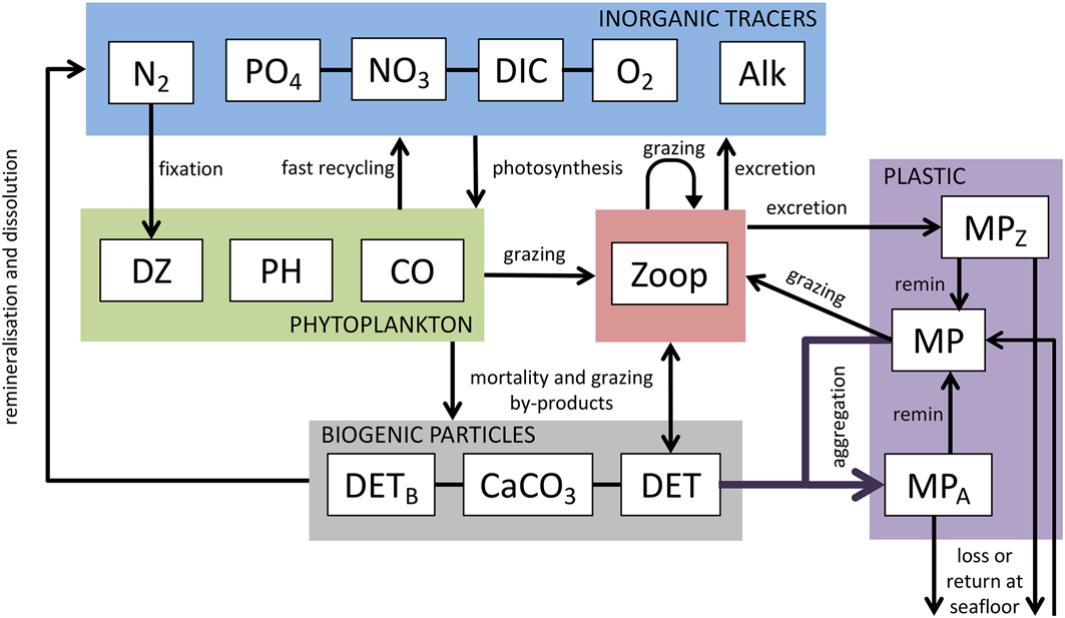
Microplastic model schematic. Marine snow is produced as a fixed fraction of the free detritus (DET) pool. MP aggregates with this marine snow, entering the $\hbox{MP}_A$ (marine snow entrained MP) pool. $\hbox{MP}_A$ held in aggregates sinks at the aggregate rate, with a fraction reaching the seafloor considered to be lost from the ocean. Detrital remineralisation releases the $\hbox{MP}_A$ from marine snow aggregates at the rate of detrital remineralisation. MP is also grazed by zooplankton and excreted into a pellet-bound $\hbox{MP}_Z$ pool. Pellet-bound $\hbox{MP}_Z$ sinks and is released back to the free MP pool at the rate of detrital remineralisation, but some is also lost at the seafloor. Details on the biogeochemical aspects of the model are previously described^43^.

For this study we use the University of Victoria Earth System Climate Model (UVic ESCM) version 2.9^40–42^. The UVic ESCM is an intermediate-complexity earth system model with a resolution of 1.8$^\circ$ latitude by 3.6$^\circ$ longitude and 19 ocean depth levels. The surface ocean level is 50 m deep. The model contains a two dimensional energy moisture-balance model of the atmosphere, as well as representations of sea ice, ocean circulation and sediments, and terrestrial carbon. The particular biogeochemical version used here includes three phytoplankton functional types, namely diazotrophs (DZ), mixed phytoplankton (PH), and small phytoplankton and calcifiers (CO)^43^. The model pre-industrial climate has been previously described^43^, as has its response to business-as-usual atmospheric $\hbox{CO}_2$ forcing^44^. The following sections describe the MP model. A model schematic is presented in Fig. 6.

### Model description

The base model^43^ was modified in order to quantify the roles of two of the three theorised biological export pathways on MP (aggregation in marine snow and zooplankton ingestion; for now, we neglect an explicit representation of biofouling^29^). We distinguish between detritus that becomes faecal pellets, and the physical aggregation of marine snow, by introducing a new faecal pellet tracer to divert 50% of zooplankton particulate losses into a separate detrital pool^27^. For simplicity, this new pellet detrital pool has the same sinking parameterisation as the original detritus. Using the same sinking rates for both detrital classes produces ocean biogeochemistry that is identical to the previously published versions of the model. In the model, plastic particles only interact passively with marine snow (they do not, for example, modify aggregate sinking rates), but they interact actively with zooplankton grazing (described below). Plastic particles have been observed to both increase and decrease the sinking rates of marine snow^21,22^ and decrease the sinking rates of faecal pellets^20,38^, but for simplicity and as a first approximation we neglect these effects in our model.

Three MP compartments are introduced; “free” (unattached) microplastic (MP), microplastic aggregated in marine snow ($\hbox{MP}_A$), and microplastic in zooplankton faecal pellets ($\hbox{MP}_Z$). All MP are considered to represent particles within a biologically active size range, but this size (and the particles’ composition) is never made explicit. These assumptions could bias modelled MP towards polymer types favoured by generic zooplankton^34^ and particle sizes in the lower end^33,34^ of the defined range of microplastic size. However, our parameter sensitivity testing in the Supplemental Information tests various fractional uptake rates that implicitly consider size and particle composition in how biologically “active” the MP pool is. As with all model ocean tracers, microplastic concentrations (MP) vary according to:

1 $$\begin{aligned} \frac{d\mathrm {MP}}{dt} = T + S(\mathrm {MP}) \end{aligned}$$

With *T* including all transport terms and *S* representing all source minus sink terms. The source and sink terms for free microplastic are:

2 $$\begin{aligned} S(\mathrm {MP}) = Emis - S(\mathrm {MP}_A) - S(\mathrm {MP}_Z) + w_p \frac{\delta \mathrm {MP} \times F_R}{\delta z} \end{aligned}$$

Microplastic is emitted to the ocean (*Emis*) along coastlines and major shipping routes using a scaling against regional $\hbox{CO}_2$ emissions (a dataset provided with the standard UVic ESCM version 2.9 package download), in order to approximate degree of industrialisation and population density in this first version of this model. The rate of emission is a proportion of the total annual plastic waste generation ($F_T$)^45^. For now, abiotic degradation of macroplastics as a source of microplastics to the ocean is neglected to keep the model simple and focus on biological transport. The MP then exchanges with the marine snow ($\hbox{MP}_A$) and zooplankton faecal pellet ($\hbox{MP}_Z$) pools. A fast particle rising rate ($w_p$) of 1.9 cm per second^46^ is prescribed to a fraction ($F_R$) of the free MP in each grid cell below the surface level as an approximation of positive buoyancy. An alternative approach would be to assign a uniform rise rate to all MP particles, and to subject the value of the rise rate to sensitivity testing. However, a weakness of this alternative approach is that the many types of plastic in the ocean have different characteristic buoyancies, which could produce unique particle pathways^18^. In this alternative approach it would be more appropriate to explicitly simulate multiple MP types in the model (which we sought to avoid in this first modelling effort for the sake of simplicity). Nevertheless, we conducted a sensitivity test using several different rise rates, and the effect of reducing the mean rise rate was similar to reducing the fraction assigned a rise rate.

In the current model version there are no abiotic breakdown rates (i.e., photo-degradation^39^) or respiration losses^47^ removing MP from circulation.

MP is modelled to aggregate in marine snow as:

3 $$\begin{aligned} S(\mathrm {MP}_A) = A_{upt} - A_{rel} - w_D\frac{\delta \mathrm {MP}_A}{\delta z} \end{aligned}$$

MP particles are taken up ($A_{upt}$) via a Monod function applied to the rate of marine snow formation (sources of detritus; $D_A$ in nitrogen units, multiplied by an aggregation fraction, $F_A$) in order to approximate an increased likelihood of MP/marine snow encounter with increasing MP concentrations that approaches a level of saturation at high MP concentrations:

4 $$\begin{aligned} A_{upt} = \frac{\mathrm {MP}}{k_P + \mathrm {MP}} \times source(D_A) \times F_A \end{aligned}$$

The uptake constant ($k_P$) is subjected to sensitivity testing, as is the fraction of marine snow aggregation ($F_A$). In this parameterisation, the aggregation of MP in marine snow represents the net uptake of MP into aggregates by both aggregation and biofouling processes. Biofouling occurs mostly in the upper 50 m^35^, which is the entire surface layer grid cell in our model. The entrainment-release cycle of biofouling is implicit in our parameterisation via the microbial loop, which is temperature-dependent. Sensitivity testing of the $k_P$ and $F_A$ parameters therefore represent testing of the net aggregation due to non-zooplankton biological aggregation effects. MP is released ($A_{rel}$) from marine snow at the rate of detrital remineralisation ($\mu _D$). This rate is temperature-dependent and results in higher rates of release in the low latitudes.

5 $$\begin{aligned} A_{rel} = \mu _D \mathrm {MP}_A \end{aligned}$$

A particle sinking term ($w_D$) applies to marine snow-associated MP, and has the same value as sinking detritus. The base unit of all MP tracers is number of plastic particles. As a first approximation we assume that all marine snow aggregates forming from free detritus have the characteristic of diatom aggregates (8.8 $\upmu$g C per aggregate^48^). Model detritus in mmol N is converted to mmol C using Redfield stoichiometry, which is then converted to $\upmu$g C to calculate the maximum number of aggregates. The maximum number of aggregates is then multiplied by the aggregation fraction $F_A$, to calculate $\hbox{MP}_A$ source and sink rates. MP is conserved for all MP tracers when surface flux balances sedimentary loss rate. What fraction of MP particles reaching the seafloor via aggregate and faecal pellet ballasting are returned to the water column ($F_B$) is tested. For simplicity and as a first approximation, detritus ballasted by calcite, and calcite^43^, are assumed to not aggregate with microplastic.

Similarly, for MP associated with zooplankton, sources and sinks are:

6 $$\begin{aligned} S(\mathrm {MP}_Z) = P_{upt} - P_{rel} - w_D\frac{\delta \mathrm {MP}_Z}{\delta z} \end{aligned}$$

The calculation of MP particle ingestion rate ($P_{upt}$) is the same as for other food sources^37^. A grazing preference ($\psi _{MP}$) for MP is subjected to sensitivity testing. This sensitivity testing implicitly examines effects such as biofouling altering the grazing preference of zooplankton for MP. It is assumed that 100% of ingested MP will be egested as faecal pellets and released ($P_{rel}$) to the “free” MP pool at the rate of faecal pellet remineralisation, with no plastic remaining in the gut and no plastic being metabolised by the zooplankton. Ingesting MP also results in a reduced zooplankton carbon uptake rate^19^, with implications for primary and export production (although, Redfield ratios are conserved). Pellet-bound $\hbox{MP}_Z$ is considered to sink at the rate of faecal pellets ($w_D$).

Plastic is eaten by zooplankton in this model. The Holling II grazing formulation^37^ is extended to include MP. Grazing of MP ($G_{MP}$) is calculated as:

7 $$\begin{aligned} \begin{aligned} G_{MP}&= \mu _Z^{max} \times Z \times \mathrm {MP}\times R_{M:P}\times R_{F:MP}\times R_{N:F}\times \psi _{MP}\\&\quad \times \,(\psi _{CO}CO+\psi _{PH}PH+\psi _{DZ}DZ+\psi _{Detr_{tot}}Detr_{tot}\\&\quad +\,psi_{Z}Z+\psi _{MP}\mathrm {MP}\times R_{M:P}\times R_{F:MP}\times R_{N:F} + k_Z)^{-1} \end{aligned} \end{aligned}$$

The maximum potential grazing rate ($\mu _Z^{max}$) is scaled by zooplankton population (*Z*) and MP availability (MP), and weighted by a food preference ($\psi _{MP}$), total prey (CO, PH, DZ, $\hbox{Detr}_{{tot}}$), and *Z* representing the food sources described in^44^ and a half saturation constant for zooplankton ingestion ($k_z$). Grazing preferences must always sum to 1 in the model, so sensitivity testing of $\psi _{MP}$ requires that all grazing preferences must also be adjusted. This is done by varying $\psi _{MP}$ but requiring $\psi _{DZ}$ always be set to 0.1 (on the basis that diazotrophs are a poor food source, and to minimize disruption to the nitrogen cycle). The remaining allowance is equally split by the other $\psi$ terms. The calculation occurs in N units, so MP is first converted to grams of MP using the MP particle-to-mass conversion of 236E3 tonnes MP = 51.2E12 particles MP ($R_{M:P}$)^4^. It is assumed that 1 g MP will roughly replace 1 g of food (at Redfield ratios; $R_{N:F}$ is the conversion from mol Food to mol N) in the zooplankton’s diet, and MP is thus converted to mmol N for the grazing calculation. However, we subject this ratio ($R_{F:MP}$) to sensitivity testing. Zooplankton uptake of plastic is therefore:

8 $$\begin{aligned} P_{upt} = \frac{G_{MP}}{R_{M:P}\times R_{F:MP}\times R_{N:F}} \end{aligned}$$

MP particles are released from faecal pellets via remineralisation, which occurs at the same rate as the remineralisation of aggregates:

9 $$\begin{aligned} P_{rel} = \mu _D \mathrm {MP}_Z \end{aligned}$$

### Model forcing

**Figure 7:**
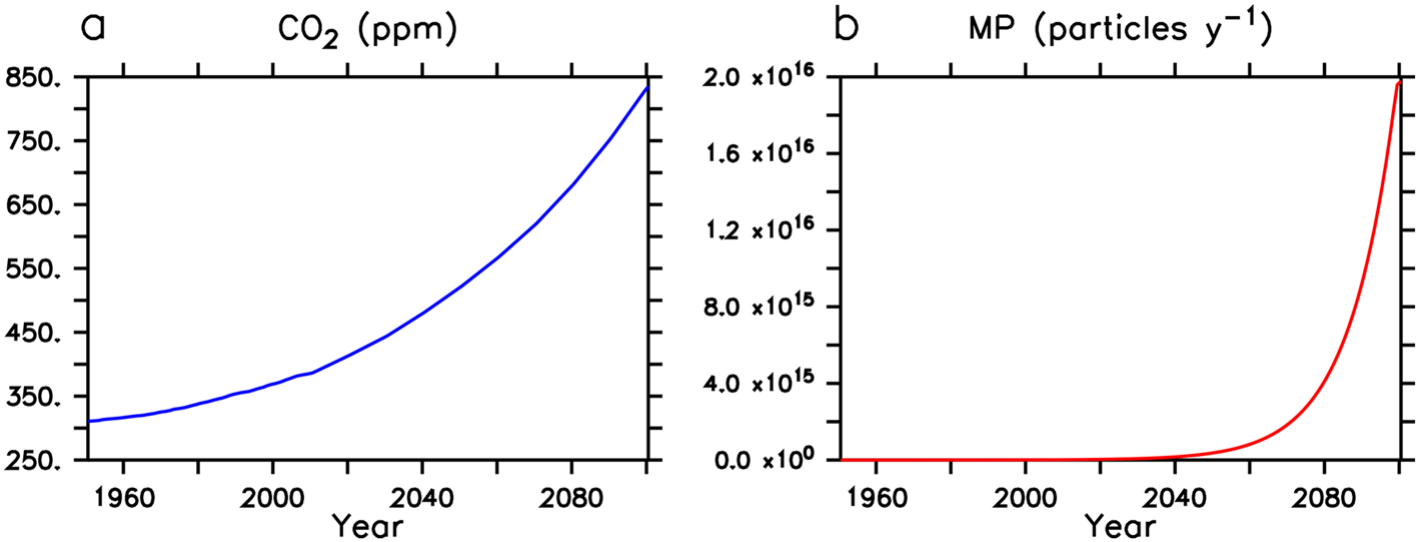
Model forcing from years 1950–2100. Atmospheric $\hbox{CO}_2$ follows RCP8.5 (panel **a**). Plastic flux into the ocean is assumed to be some fraction of the total historical and projected plastic waste generation estimate (panel **b**), with a continuing rate of increase of 8.4% per year^45^, converted to MP particles using a mass conversion^4^. Previous estimates of actual total plastic mass flux into the ocean is only about 4% of the total plastic waste generation^30^, with the MP fraction being a small proportion of that.

The model was integrated at year 1765 boundary conditions (including agricultural greenhouse forcing and land ice) for more than 10,000 years until equilibration was achieved. From year 1765 to 1950, historical $\hbox{CO}_2$ concentration forcing, and geostrophically adjusted wind anomalies are applied. From 1950 to 2100 the model is forced with a combination of historical $\hbox{CO}_2$ concentration forcing (to 2000) and a business-as-usual high atmospheric $\hbox{CO}_2$ concentration projection RCP8.5^49,50^. MP emissions start from 2 million metric tonnes in year 1950 (a total plastic waste generation estimate^45^), increasing at a rate of 8.4% per year. $\hbox{CO}_2$ and MP forcing is summarized in Fig. 7. It has been estimated that about 4% of total plastic waste generated enters the ocean^30^, but that the microplastic mass found at the sea surface represents only about 1% of the annual plastic input to the ocean^4^. We test a range of input fractions (see Table 2), after applying a mass conversion from tonnes to number of MP particles^4^. Using a considerable over-estimation of MP pollution rate also implicitly accounts for abiotic degradation of larger plastics.

**Table 2:** Microplastic model parameters and range tested.

Parameter	Abbreviation	Units	Range
Input fraction of total plastic waste generation	$F_T$	–	0–1
Fraction of MP that rises	$F_R$	–	0–0.1
Fraction of marine snow that aggregates	$F_{A}$	–	0–1
MP marine snow uptake coefficient	$k_P$	Particles $\hbox{m}^{-3}$	0–1000
Fractional seafloor MP return	$F_B$	–	0–1
Zooplankton MP grazing preference	$\psi _{MP}$	–	0.1–0.3
Food/MP substitution ratio	$R_{F:MP}$	g Food: g MP	0.5–1.5

### Experimental setup

A 700-member Latin Hypercube^51^ was used to test the microplastic parameter space of the model using the forcing described in the previous section. While biological model parameters might also influence microplastic uptake and transport, we limited our initial tests to the new parameters introduced above. A range of values was prescribed to the parameters listed in Table 2, in which the parameter space was randomly sampled with a normal distribution. The objective was to see what can be learned about plastic accumulation in the ocean, when very little is known about plastic/particle interactions and basic processes are still poorly understood. An analysis of the full Latin Hypercube parameter search is provided as Supplemental Information.

We adopted an incremental approach to increasing model complexity. We started with a control Hypercube where biology was not allowed to take up plastic, in order to first test the physical parameters ($F_T$ and $F_R$, the fraction of total annual plastic produced entering the ocean as MP, and the fraction assigned a rise rate, respectively). One hundred simulations were performed in this configuration, with the results analysed in the Supplemental Information. We next included passive plastic aggregation in marine snow (MP plus the $\hbox{MP}_A$ tracer) in a 300 simulation Hypercube, spread across the $k_P$ (marine snow uptake coefficient) parameter space (0–1, 1–100, 100–1000 particles $\hbox{m}^{-3}$, each with 100 Hypercube simulations) in a normal distribution. These 300 simulations explored the 5 relevant MP model parameters: $F_T$, $F_R$, $F_A$ (marine snow aggregation fraction), $k_P$, and $F_B$ (fractional return to ocean at the seafloor). These results are also provided in the Supplemental Information. Finally, we added active zooplankton-associated plastic (MP, plus $\hbox{MP}_A$ and $\hbox{MP}_Z$ tracers) as a third 300-individual Hypercube set. This third Hypercube is similarly split across the $k_P$ parameter space in a normal distribution, but with the addition of grazing parameters $\psi _{MP}$ (MP grazing preference) and $R_{F:MP}$ (the food to MP substitution ratio; 7 parameters in total).

## Supplementary information

Supplementary material 1.

## Data Availability

Model output and postprocessing scripts are available at: https://hdl.handle.net/20.500.12085/a3c47dd2-ee93-475f-bca8-e7f58d3c2fec. Model code is available from the first author upon request.
