# Supplementary material for: The global biological microplastic particle sink
Source: Sci Rep. 2020 Oct 7;10:16670. doi: 10.1038/s41598-020-72898-4 (PMC7542466; doi:10.1038/s41598-020-72898-4)
Supplement: Supplementary file 1 — Supplementary material 1. [file 41598_2020_72898_MOESM1_ESM.pdf]

Supplementary information for “The global biological microplastic particle sink”

K Kvale[1,*], AEF Prowe[1], C-T Chien[1], A Landolfi[1,2], and A Oschlies[1]

[1]GEOMAR Helmholtz Centre for Ocean Research, West shore campus, Duesternbrooker Way 20, D-24105 Kiel, Germany
[2]ISMAR-CNR, Via Fosso del Cavaliere, 100, 00133, Roma, Italy
[*]kkvale@geomar.de

Parameter sensitivity results

If the global ocean is accumulating MP, then the rate of pollution must be overwhelming the ability of biology and physical degradation to remove it. Despite little being known regarding the mechanisms of biological MP uptake, parameter testing still reveals some interesting aspects of MP accumulation in the global ocean. Note all the simulations presented here include both increasing atmospheric CO_2 concentrations, as well as increasing rates of plastic pollution, and cannot be considered steady-state.

MP with no biological uptake stays in the upper ocean

Two parameters, the fraction of total plastic waste generation that enters the ocean as MP (F_T) and the fraction of MP prescribed a rising rate (F_R) were tested with Latin Hypercube sampling of the parameter space given in Table 1 of the main paper. It was found that simulations in which high fractions were assigned a rise rate were numerically unstable due to the very fast rise rate (F_R greater than about 0.05, or more than 5% of the MP having positive buoyancy). In this mass-conserving model, numerical instability results in unrealistically large positive, as well as large negative MP concentrations, rendering the simulations unusable. It was also found that none of the explored MP metrics related to fractional storage of the MP inventory were sensitive to F_T, although absolute quantities such as total ocean MP inventory (second row, Fig. S1) and maximum surface concentration in gyres (Fig. S2) are.

Nearly all values of F_R retained greater than 90% of the total emitted MP inventory in the top 100 meters of the ocean (Fig. S1), with the remaining plastic found above 500 m depth (Fig. S1). In all simulations, global MP accumulation below 500 m depth is negligible relative to the total inventory. No dependency on time is found in the depth at which the inventory is held (compare blue circles; year 2010, versus red circles; year 2100). This suggests that for the buoyant and weakly buoyant fraction of MP in the real ocean, in the absence of biological uptake, greater than 90% of the particle load can be expected to remain in the upper 100 m into the future. However, MP particles with lower buoyancy and passively tracing can be expected to have a presence in the 100-500 m depth zone, with as much as 25% of the total passive inventory residing there, now and into the future.

Latitudinal MP distributions are sensitive to the fraction assigned a rise rate

Latitudinal distribution is also sensitive to F_R, with larger fractions of MP rising associated with lower inventory retention between 40 S and 40 N (Fig. S1). The rising MP escapes into the northern hemisphere mid-latitudes (40-60 N; particularly around the North Sea, with both North Atlantic and Arctic accumulation showing sensitivity to F_R; Fig. S3). Our results are generally consistent with the findings of[1] that neutrally buoyant plastics accumulate in the low latitudes; any differences may be due to methodology (differences in plastic input mask, applied buoyancy assumptions, physical circulation). Accumulation of MP in the major ocean gyres occurs in our model (three samples are shown in Fig. S3 which include low, moderate, and high F_R values of 0.01, 0.03, and 0.05). Figure S2 gives resulting maximum surface concentrations for each major ocean gyre at year 2010. But the greatest maximum surface concentrations generally occur along coastlines and in semi-enclosed seas, rather than in the gyres (which does not disagree with, e.g.[2] finding MP concentrations of about 2 particles per m^3 in the open ocean around Japan, which is roughly double the observed concentration in the North Pacific Gyre. But also, the

models using F_R values greater than 0.04 produce the highest maximum concentrations in the North Sea. This suggests that the more buoyant MP is transported by boundary currents, which favours export to the mid latitudes, while the more neutrally buoyant MP follows more complex pathway(s). No significant plastic accumulation occurs in any simulations in the Southern Ocean, and accumulation in the Arctic above 60 N represents less than 2.5% of the inventory in all simulations. Interestingly, a decrease in F_R increases the representation of MP in the Arctic, which suggests deposition there follows a deeper and more passive pathway. Likewise, maximum surface gyre concentration in the Indian Ocean displays a sensitivity to both F_R and F_T (Fig. S2), which suggests complex accumulation pathways for this basin.

A notable dependency on time (climate change + MP emissions forcing) is found in the decreasing fraction of the total inventory residing in the low latitudes into the future, for the larger F_R values. A shift of the inventory to the middle latitudes occurs in these simulations (not shown), overwhelmingly in the North Atlantic. This occurs despite the slowing down of the Atlantic Meriodional Overturning Circulation (AMOC) over this time period (not shown), because the surface currents speed up and more effectively transport the buoyant and semi-buoyant fractions. As a result, an increasing proportion of the total MP inventory is held in the North Sea and in the coastal zones of north-western Europe.

MP depth distributions are sensitive to marine snow uptake

Aggregation of MP with marine snow is next introduced to the model. Intuitively, global MP inventory and maximum gyre surface concentration are still sensitive to the fractional MP input into the ocean (F_T; Figs. S4 and S5). Our simulations show that introduction of marine snow uptake both significantly reduces maximum surface concentration in the gyres (particularly the North Atlantic, North Pacific, and Indian Ocean; Fig. S5) and can produce significant MP accumulation in the deep ocean (Fig. S4). The inventory fraction held below 500 m depth varies between 0 and 50% (two extreme points excepted), with generally greater proportions of MP assigned a rise rate resulting in generally smaller fractions held at depth (with exceptions; Fig. S4). However, deep ocean inventory fraction is also sensitive to k_P (efficiency of marine snow uptake), with inefficient uptake (high values of k_P) resulting in generally less MP at depth. This parameter relationship is also seen in the average depth of the maximum water column concentration (top row, Fig. S4), with increasingly efficient MP uptake by marine snow resulting in a deepening of particle maxima. However, efficient uptake of MP by marine snow must also include a low fraction of MP assigned a rise rate (less than 2.5%), in order to produce a globally significant deepening of particle maximum concentration.

Likewise, surface and sub-surface MP storage is still sensitive to the fraction MP assigned a rise rate (F_R), but is also sensitive to the marine snow/MP uptake coefficient (k_P; Fig. S4). More efficient uptake by marine snow (low k_P) results in a greater spread of potential fractional storage in every depth range; 0-100% in the upper 100 m, 0-60% between 100-500 m, and 0-50% below 500 m depth. The temporal dependency in fractional storage moving to shallower depths seen in Fig. S4 is due to the decline in detrital export production with climate warming, which reduces the detritus available for transporting MP to depth in all simulations.

Marine snow uptake reduces the possible range of MP inventory in the low latitudes

Despite latitudinal distribution of MP inventory now showing only weak sensitivity to the fraction prescribed a rise rate, and marine snow aggregation uptake parameters (Fig. S4), the most obvious difference between simulations with and without marine snow MP uptake is the absence of any parameter space including significant inventory loss to the middle latitudes when MP entrains with marine snow. Marine snow is an effective "trap"; only in 3 models does the fractional inventory held within the low latitudes drops below 70% when biological uptake is applied. "Free" MP accumulated in the North Sea in certain parameter combinations when biological uptake was not considered, which decreased the low latitude inventory to almost nothing. Our simulations of marine snow aggregation also reveal accumulation in the North Sea, and a mid-latitude fractional inventory that compensates the low-latitude fractional inventory, but within a lower and narrower range (0-30% in the North Sea) across the range of parameters tested. We lastly find no consistent dependence on time with respect to latitudinal fractional MP distribution, when marine snow is included in the simulations. These results suggest that the transport of MP by marine snow distributes the MP vertically in the water column, reducing the exposure of MP to surface boundary currents and resulting in a more diverse pattern of transport than is achieved by physical transport alone.

In our model, MP storage in the polar regions is weakly sensitive to the application of marine snow uptake and transport (Fig. S4). In the Arctic, the fraction held above 60 N is not greater than 3% of the total inventory (with one exception), but the number of simulations that show a fractional inventory greater than 0 has increased. And, in the Southern Ocean, no significant accumulation occurs for the range of parameters tested. This result is in contrast to the sub-surface transport model of[3] and is likely due to the different method of applying MP pollution- we found significant Southern Ocean pollution in sensitivity tests in which a spatially-uniform MP input was applied (as they did), but none when pollution occurs mostly along coasts.

ID	MCD	MP (part.$\times 10^{13}$)	F_T	F_R	F_B	F_A	k_P	ψ_{MP}	$R_{F:MP}$
**100 (Lo)**	272	1.260	0.137	0.003	0.132	0.003	0.424	0.260	1.029
108	33	2.890	0.290	0.041	0.109	0.0003	76.271	0.232	1.173
167	33	3.860	0.360	0.023	0.136	0.020	87.691	0.106	0.789
171	67	2.150	0.275	0.037	0.067	0.037	53.833	0.268	0.630
198	58	0.114	0.006	0.089	0.079	0.062	74.350	0.142	1.303
216	39	3.590	0.203	0.044	0.789	0.081	483.486	0.110	1.387
**219 (Hi)**	76	6.220	0.329	0.074	0.888	0.098	833.290	0.132	1.489
224	38	2.370	0.199	0.046	0.265	0.087	429.623	0.116	0.722
234	36	0.822	0.039	0.028	0.925	0.016	501.988	0.152	1.302
256	42	0.627	0.052	0.054	0.260	0.036	944.168	0.182	0.540
**275 (Med)**	101	3.220	0.276	0.011	0.528	0.092	615.508	0.193	0.993
281	42	4.510	0.220	0.020	0.959	0.003	104.312	0.196	0.854
282	37	0.600	0.040	0.028	0.371	0.013	386.028	0.167	1.207
285	56	4.620	0.261	0.066	0.847	0.074	344.654	0.103	0.912

Table S1. Numerically stable Latin Hypercube simulations in which total ocean MP pollution rates are limited to 40% or less of the annual total plastic waste generation and marine snow aggregation rates are less than 10%. Simulation ID is given in the left column, maximum concentration depth is given in meters in the second column, total particle inventory in year 2010 is given in the third column, and plastic model parameter values are given in the rest of the table. Simulations analysed in the main text are shown in boldface with their corresponding names.

Zooplankton faecal pellets transport MP to the deep ocean

Zooplankton consumption of MP increases the number of simulations with particle maximum concentrations below the surface (Fig. S6). Potential maximum surface concentrations in the gyres are likewise decreased (Fig. S7). High surface concentrations in the gyres are generally associated with higher pollution rates (F_T) and greater fractions assigned a rise rate (F_R), but there is no clear relationship between fractional storage above 100 m depth and any single biological uptake parameter. The addition of zooplankton grazing to the hypercube reduces the possible fractional storage above 100 m depth to less than 70%, now found to be in the same possible range as the sub-surface (0-60% between 100-500 m depth), with reduced fractional storage in both of these zones for the neutrally buoyant fraction. The potential range of fractional inventory held below 500 m depth increases to 10-90% at year 2010, indicating zooplankton faecal pellets are a potentially significant exporter of MP to the deep ocean, despite there being no clear relationship with grazing parameters. The temporal sensitivity in fractional storage with depth in simulations with zooplankton pellet MP transport has the same driver as that in the marine snow-only simulations; climate warming-induced reductions in export production reduces MP transport out of the surface ocean.

The application of zooplankton faecal pellet MP transport has no significant additional effect (for the parameter range tested) on polar MP storage, now and into the future. MP inventory in the Arctic is never greater than 3% of the global total. However, fractional storage in the Southern Ocean shows some high values (greater than 5%) for some parameter combinations when zooplankton grazing is included.

Reducing the uncertainty window in our simulations

The results presented above cover the whole model parameter space from no biological uptake and unrealistically low MP emissions rates to high rates of biological uptake and unrealistically high MP emissions rates. It is challenging to reduce the parameter uncertainty, given how poorly MP distributions across the global ocean are known. However, a subset of our simulations proved to have numerical instabilities, and removing these (based on the presence of large negative concentrations and the total ocean positive MP inventory at 2010 being greater than the time-integrated total ocean pollution rate) reduced the zooplankton grazing and marine snow aggregation sample size from 300 to 132 individuals. Additional filtering of the simulations to omit all those using MP input rates greater than 40% of global plastic aste generation[4] (allowing for an order-ten overestimate of plastic entering the ocean as plastic inputs might be underestimated[5]), and marine snow aggregation fractions greater than 10% (based on[6] estimating open ocean rates of 3-5% but[7] demonstrating enhanced marine snow formation rates in the presence of MP) reduces the sample size to 14 individuals (Table S1; Fig. S8).

Encouragingly, all filtered simulations have year 2010 total MP particle inventories that roughly approximate the[8] estimate of 3.02E12 (which considered the surface mixed layer only and anticipated a one-to-two orders-of-magnitude higher total particle count than observed). These filtered simulations contain relatively low fractions assigned a rise rate, ranging from 0.3-8.9%. In our formulation, variability in the fraction given a rise rate reflects differences in the bulk composition of the

MP, with relatively less, or more, buoyant material in the mix. Rates of seafloor return vary across the parameter space, from 6.7-96%. The moderate and high rates of seafloor return (greater than 10% in all but two simulations) are interesting in the context of the observation that sedimentary MP samples have a distinctly smaller size distribution than water column MP, which suggests a size-selection mechanism for sedimentary sequestration[9].

The average zooplankton MP grazing preference in our filtered samples is 0.169, which represents a slight preference for organic food sources. The model does not explicitly simulate reasons for food preference; e.g., polymer type, MP particle size, or particle "confusion". The model also does not simulate multiple trophic levels of zooplankton, which might also affect zooplankton grazing preferences[10]. While some zooplankton have been observed to ingest MP, others reject it[11], therefore a single globally-appropriate coefficient is highly uncertain. Equal weighting of food preferences of all food sources other than diazotrophs would be a ψ value of 0.18; 8 of our simulations in Table S1 fall below this threshold, and 6 above. Lastly, the ratio of food:MP substitution in the 14 simulations is also spread across the tested parameter space, with half of them exceeding a ratio of 1. That 1 gram of plastic can substitute in the zooplankton's diet for e.g., 1.3 grams of food, is consistent with a reduction in feeding efficiency that might arise due to gut blockage[11] or other stressors only accounted for implicitly in our model. However, only weak relationships with our applied metrics mean that none of the Latin Hypercube samples can be discounted on the basis of their grazing parameters.

Only one of the filtered simulations (Test100) has a marine snow MP uptake coefficient less than 1 (that is, efficient uptake which only implicitly represents the relevant processes such as particle characteristics). Efficient uptake in this model is compensated by a low marine snow aggregation rate, and the lowest fractional rise rate, both of which work to reduce MP exposure to marine snow aggregates. The other 13 tests have uptake coefficient values 2-3 orders of magnitude greater (e.g., inefficient uptake). Experiments conducted in controlled conditions document high uptake rates[7,12], however the global surface removal budget calculated by[5] suggests globally inefficient MP removal by marine snow. A suitable parameter value (and even a suitable parameterisation!) for efficiency in the real ocean carries with it perhaps the greatest uncertainty of the seven parameters tested.

The 13 samples with inefficient uptake coefficients display shallower maximum particle concentrations, globally averaged at around 50 meters depth, as opposed to 272 meters for Test100 (Table S1 and Fig. S8). However, depth profiles, averaged both globally and by major ocean basin, display distinctly different concentration curves that are not easily explained by single model parameters (Fig. S9; note the different axes limits for MP_A). These profiles instead demonstrate the relative strength of, and complex interaction between, MP sources and sinks. E.g., relative differences in total MP inventory (a function of input and seafloor return) cannot explain relative differences in MP concentrations with depth, and a more efficient marine snow uptake coefficient does not always result in greater MP_A concentrations. According to our MP profiles, a maximum MP concentration at the surface does not necessarily contradict the observations of[8] and[13] of a "missing" quantity of MP at the surface because the sub-surface inventory can be significant. Figure S9 shows local particle minima in the "free" MP compartment around 100 m depth, where the MP_Z concentrations reach their maxima. MP concentrations increase just below the MP_Z particle maxima, as MP is released from sinking pellets through organic remineralisation.[14] recently found MP concentration maxima between 200-600 m in a regional survey of the open ocean. Such a particle profile is tantalizing similar to[15] reporting an organic particle maxima at 300-600 m depth along the Equator as well as[16] observing a MP particle minima at around 100-200 m depth, which implies significant uptake and transport of MP by marine snow (and, as our study suggests, pellets) might also be happening in real-world conditions. If future oceanographic surveys can demonstrate a globally robust MP particle maxima within the depth range of the organic particle maxima, our modelling suggests this is a key indicator of the efficiency of MP entrainment in biological particles.

In the main text we focus on 3 representative samples from Table S1; Test100 (labelled TestLo), Test219 (labelled TestHi), and Test275 (labelled TestMed). TestLo is chosen to represent a model that produces low "free" MP surface concentrations. It has efficient marine snow uptake of MP, and is characterised by a moderate total inventory and the deepest particle maximum. TestHi is chosen because it has the highest "free" MP surface concentrations. It is also characterised by the greatest total MP inventory, and uses efficient zooplankton uptake of MP. TestMed is a moderate configuration, with intermediate surface concentrations in all MP compartments, and a moderate total MP inventory. These three samples are chosen to represent the range of surface removal potential for the 14 member Hypercube subset. However, newly published MP concentration profiles from the North Pacific Gyre favours those simulations that produce power law-shaped profiles[16] that also include local minima in the upper 50-200 meters, as well as local maxima around 200-500 meters depth. Of our 3 selected samples, Test219 (TestHi) produces the MP concentration profile closest to this new dataset. In the 14 member subset shown in Fig. S9, Test108, Test219, Test224, and Test285 all produce similarly logarithmic MP concentration profiles.

References

1. Mountford, A. S. & Morales Maqueda, M. A. Eulerian modeling of the three-dimensional distribution of seven popular microplastic types in the global ocean. *J. Geophys. Res. Ocean.* **124**, 8558–8573, DOI: 10.1029/2019JC015050 (2019).

https://agupubs.onlinelibrary.wiley.com/doi/pdf/10.1029/2019JC015050.

2. Isobe, A., Uchida, K., Tokai, T. & Iwasaki, S. East asian seas: A hot spot of pelagic microplastics. *Mar. Pollut. Bull.* **101**, 618 – 623, DOI: https://doi.org/10.1016/j.marpolbul.2015.10.042 (2015).

3. Wichmann, D., Delandmeter, P. & van Sebille, E. Influence of near-surface currents on the global dispersal of marine microplastic. *J. Geophys. Res. Ocean.* **124**, 6086–6096, DOI: 10.1029/2019JC015328 (2019). https://agupubs.onlinelibrary.wiley.com/doi/pdf/10.1029/2019JC015328.

4. Jambeck, J. R. *et al.* Plastic waste inputs from land into the ocean. *Science* **347**, 768–771, DOI: 10.1126/science.1260352 (2015). https://science.sciencemag.org/content/347/6223/768.full.pdf.

5. Kvale, K. F., Friederike Prowe, A. E. & Oschlies, A. A critical examination of the role of marine snow and zooplankton fecal pellets in removing ocean surface microplastic. *Front. Mar. Sci.* **6**, 808, DOI: 10.3389/fmars.2019.00808 (2020).

6. Shanks, A. & Trent, J. Marine snow - sinking rates and potential role in vertical flux. *Deep. Res. Part A-Oceanographic Researcg Pap.* **27**, 137–143, DOI: 10.1016/0198-0149(80)90092-8 (1980).

7. Michels, J., Stippkugel, A., Lenz, M., Wirtz, K. & Engel, A. Rapid aggregation of biofilm-covered microplastics with marine biogenic particles. *Proc. Royal Soc. B: Biol. Sci.* **285**, 20181203, DOI: 10.1098/rspb.2018.1203 (2018). https://royalsocietypublishing.org/doi/pdf/10.1098/rspb.2018.1203.

8. Eriksen, M. *et al.* Plastic pollution in the world's oceans: More than 5 trillion plastic pieces weighing over 250,000 tons afloat at sea. *PLOS ONE* **9**, 1–15, DOI: 10.1371/journal.pone.0111913 (2014).

9. Peng, X. *et al.* Microplastics contaminate the deepest part of the world's ocean. *Geochem. Perspectives Lett.* **9**, 1–5, DOI: http://dx.doi.org/10.7185/geochemlet.1829 (2018).

10. Sun, X. *et al.* Ingestion of microplastics by natural zooplankton groups in the northern south china sea. *Mar. Pollut. Bull.* **115**, 217 – 224, DOI: https://doi.org/10.1016/j.marpolbul.2016.12.004 (2017).

11. Cole, M. *et al.* Microplastic Ingestion by Zooplankton. *Environ. Sci. & Technol.* **47**, 6646–6655, DOI: 10.1021/es400663f (2013).

12. Moehlenkamp, P., Purser, A. & Thomsen, L. Plastic microbeads from cosmetic products: an experimental study of their hydrodynamic behaviour, vertical transport and resuspension in phytoplankton and sediment aggregates. *ELEMENTA-SCIENCE OF THE ANTHROPOCENE* **6**, DOI: 10.1525/elementa.317 (2018).

13. Cózar, A. *et al.* Plastic debris in the open ocean. *Proc. Natl. Acad. Sci.* **111**, 10239–10244, DOI: 10.1073/pnas.1314705111 (2014). https://www.pnas.org/content/111/28/10239.full.pdf.

14. Choy, C. A. *et al.* The vertical distribution and biological transport of marine microplastics across the epipelagic and mesopelagic water column. *Sci. Reports* **9**, 7843, DOI: 10.1038/s41598-019-44117-2 (2019).

15. Kiko, R. *et al.* Biological and physical influences on marine snowfall at the equator. *Nat. Geosci.* **10**, 852–858, DOI: 10.1038/ngeo3042 (2017).

16. Egger, M., Sulu-Gambari, F. & Lebreton, L. First evidence of plastic fallout from the North Pacific Garbage Patch. *Sci. Reports* **10**, 7495, DOI: 10.1038/s41598-020-64465-8 (2020).

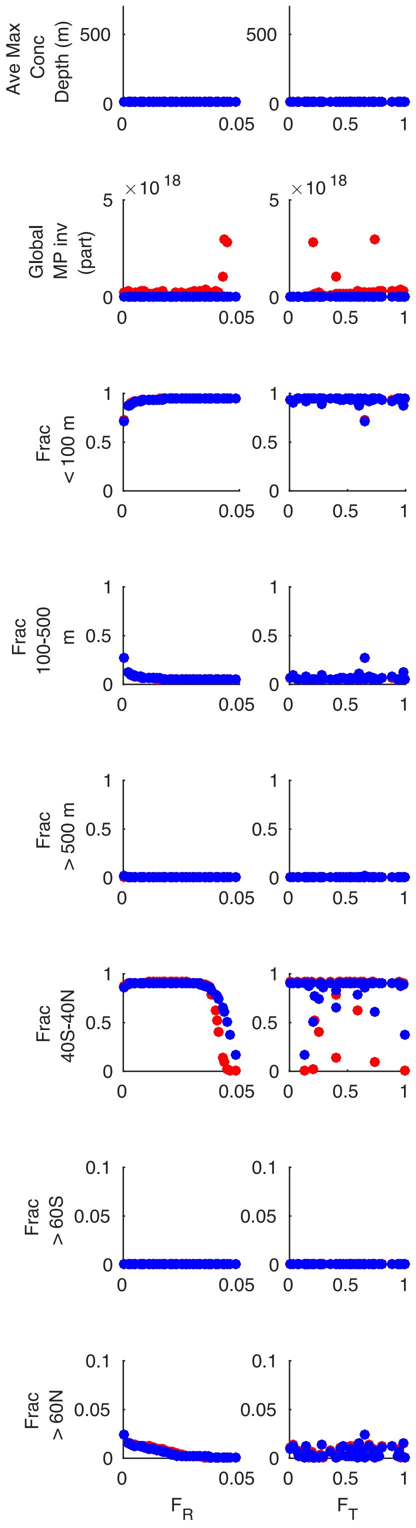

Figure S1. Fraction of the total MP inventory held within various ocean zones as a function of proportion of MP assigned a rise rate (left column) and proportion of total plastic input entering as MP (right column) at year 2010 (blue circles) and year 2100 (red circles).

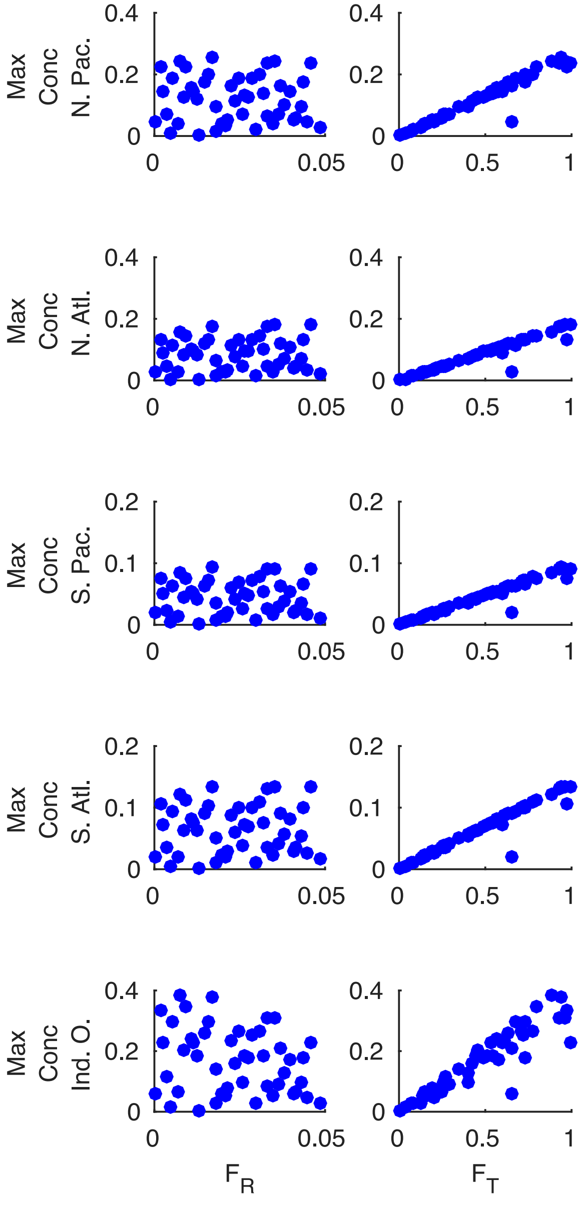

Figure S2. Maximum surface grid cell concentration (particles per m^3) in the gyre of each ocean region, as a function of proportion of MP assigned a rise rate (left column) and proportion of total plastic input entering as MP (right column) at year 2010.

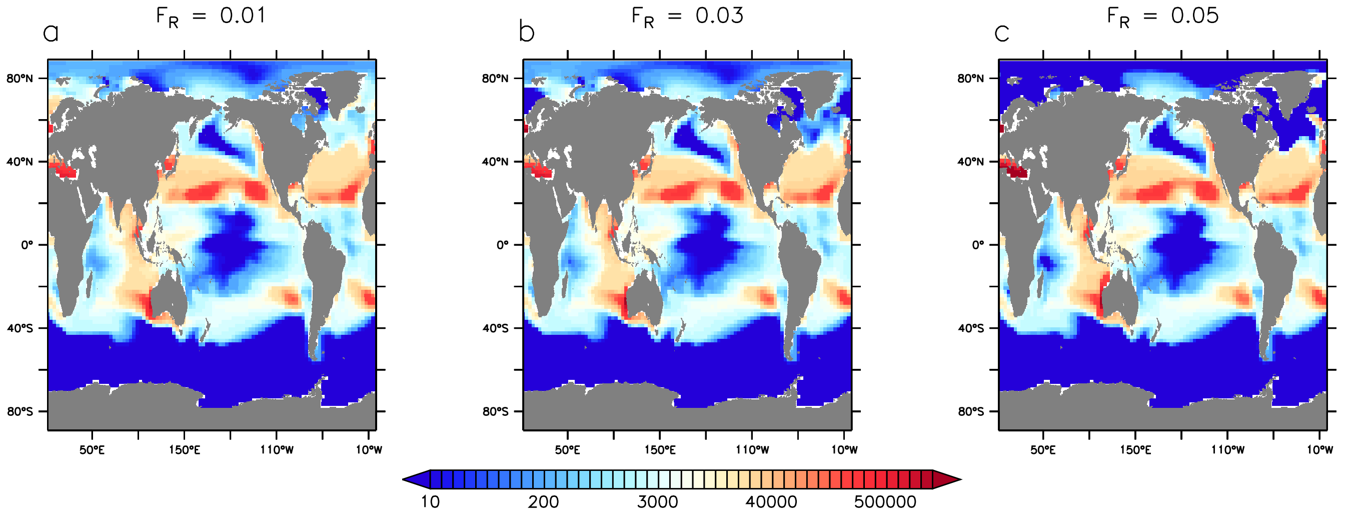

Figure S3. MP surface concentration (particles per km^2 in the top 1 meter) samples at year 2010, from models to which low (panel a), moderate (panel b) and large (panel c) F_R values are applied.

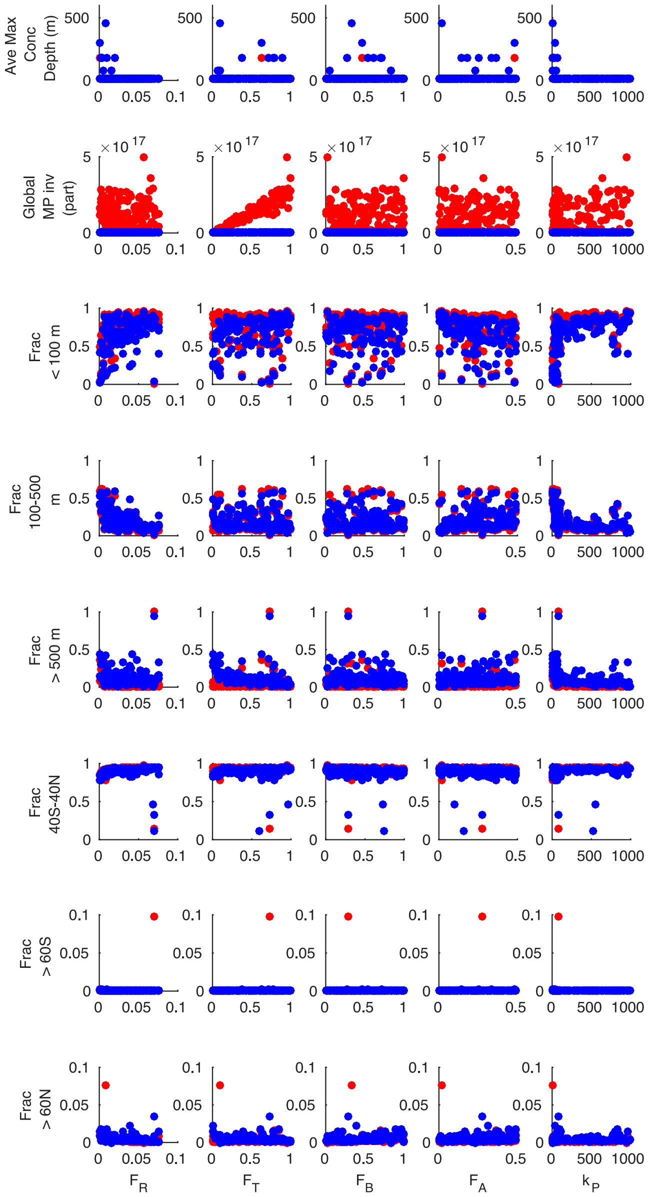

Figure S4. Fraction of the total MP inventory held within various regions as a function of MP model parameters at year 2010 (blue circles) and year 2100 (red circles). Results represent the marine snow aggregation simulations.

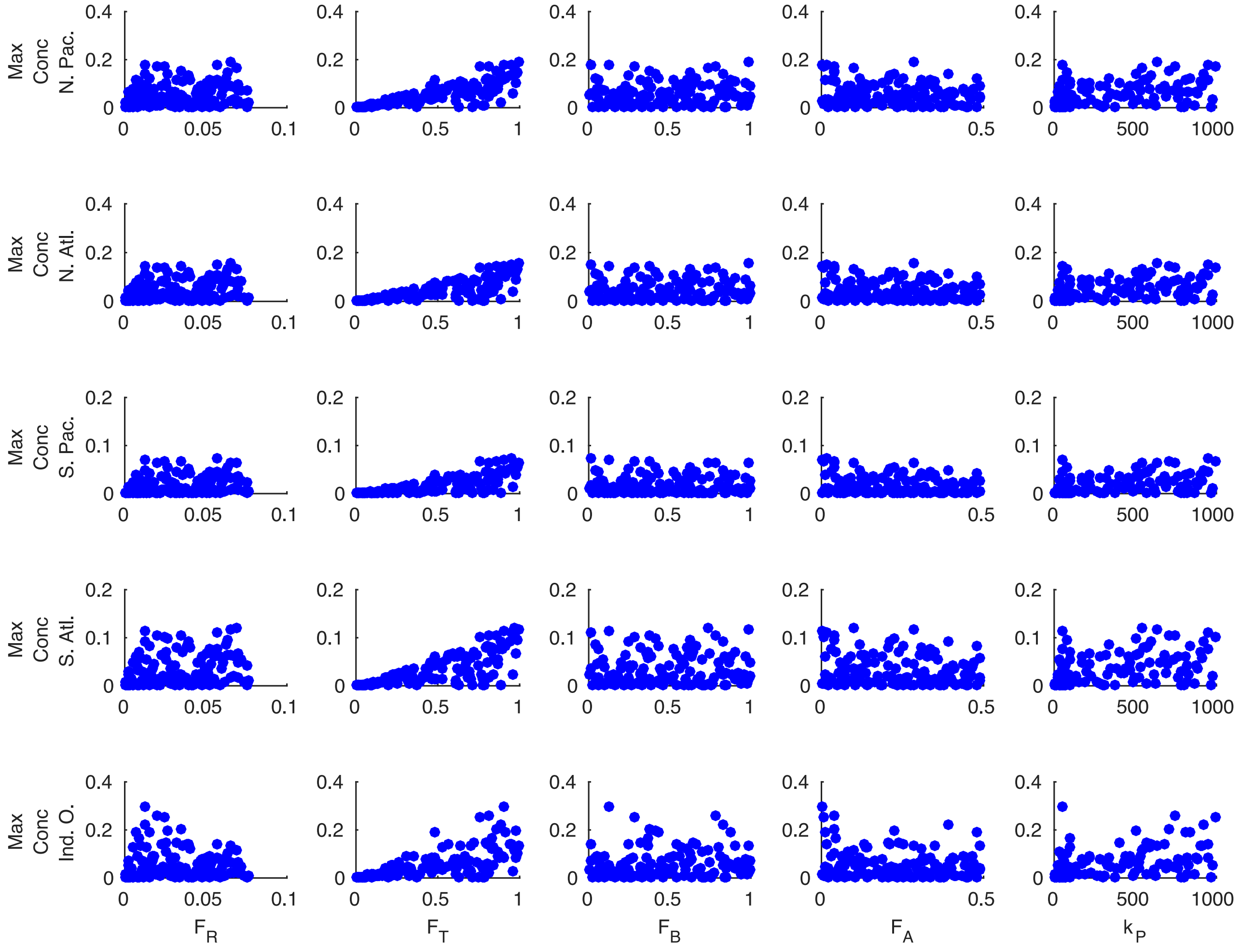

Figure S5. Maximum surface grid cell concentration (particles per m^3) in the gyre of each ocean region, as a function of MP model parameters at year 2010. Results represent the marine snow aggregation simulations.

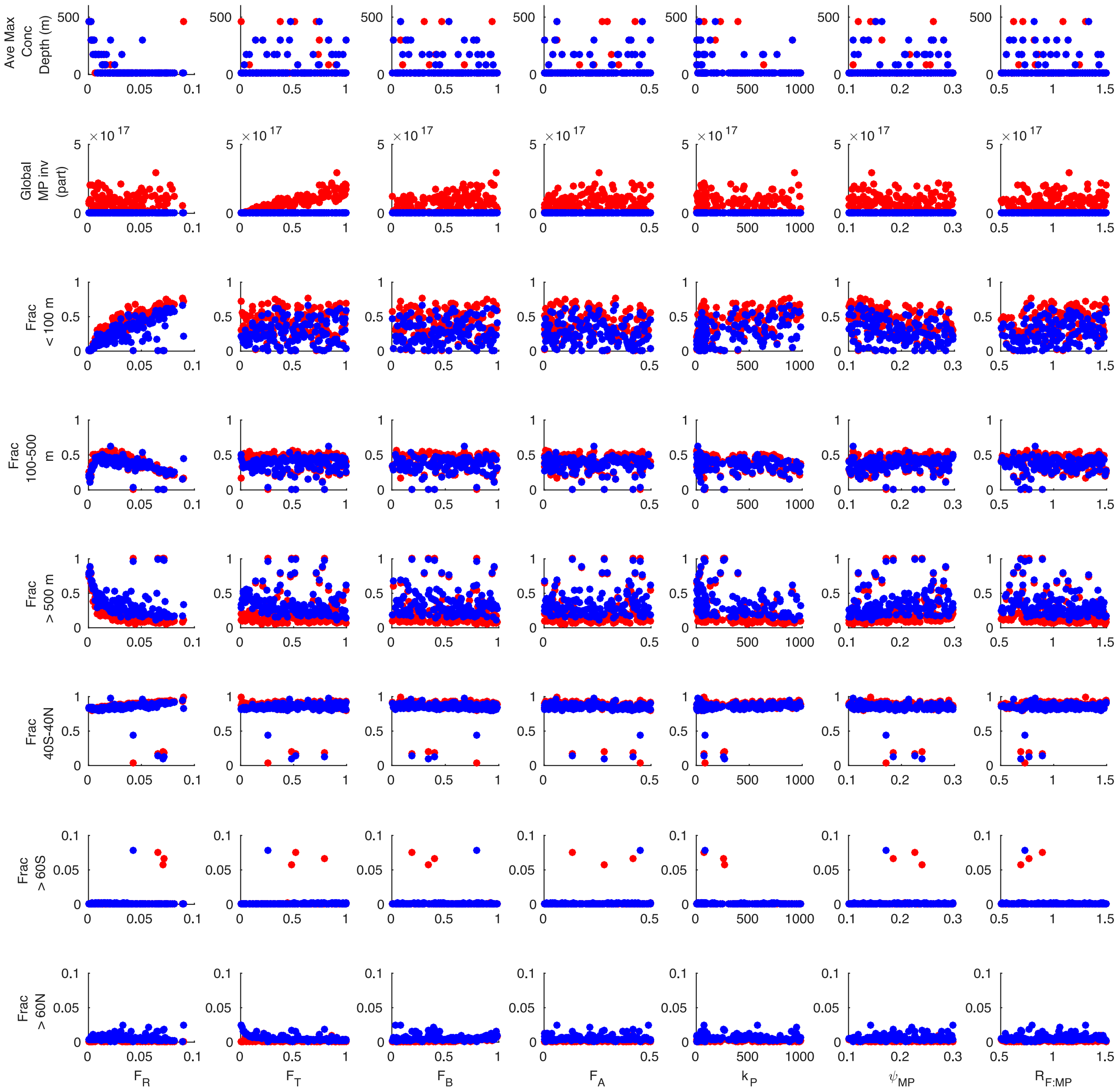

Figure S6. Fraction of the total MP inventory held within various regions as a function of MP model parameters at year 2010 (blue circles) and year 2100 (red circles). Results represent the simulations including both marine snow and zooplankton consumption.

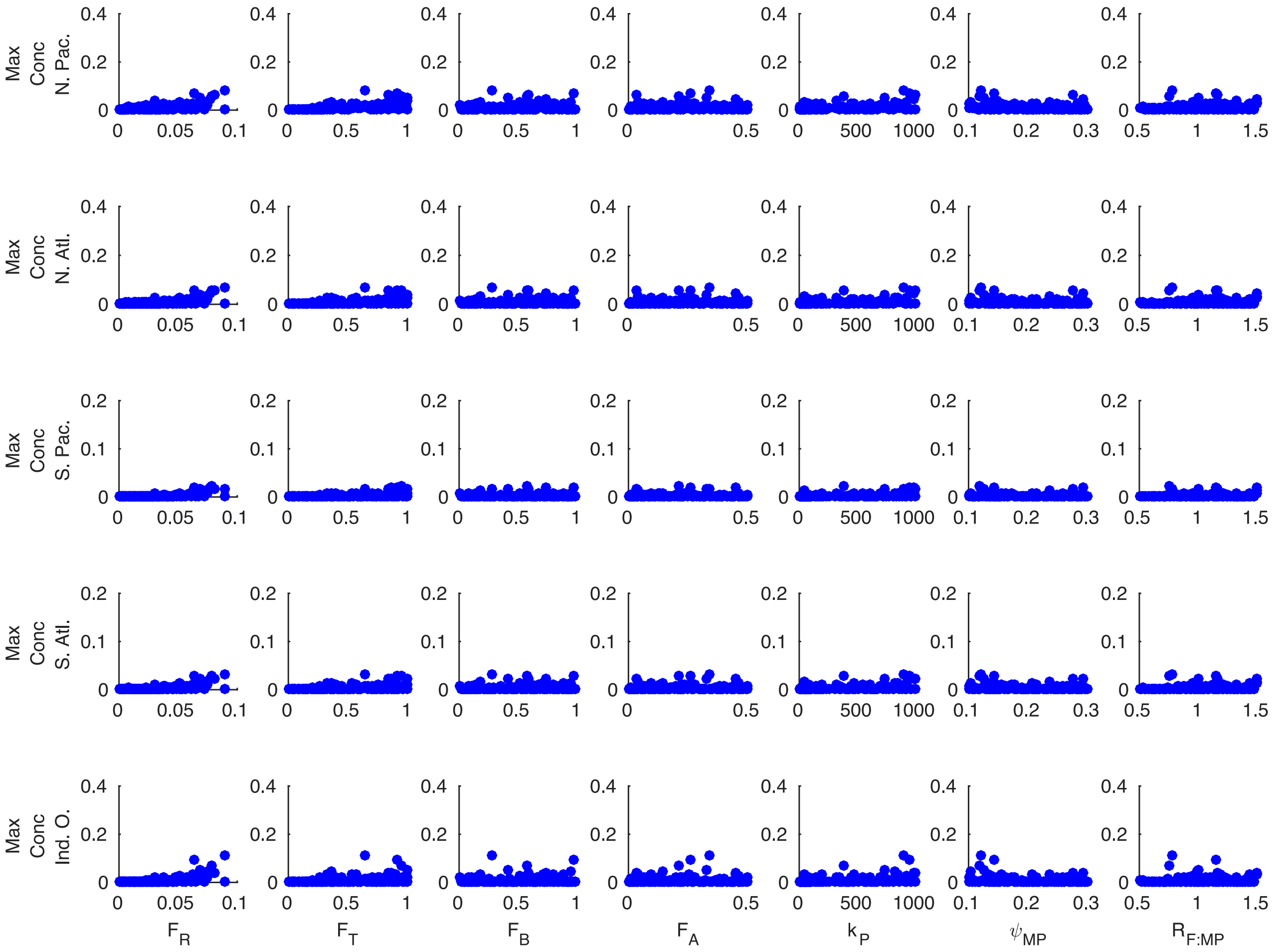

Figure S7. Maximum surface grid cell concentration (particles per m^3) in the gyre of each ocean region, as a function of MP model parameters at year 2010. Results represent the simulations including both marine snow and zooplankton consumption.

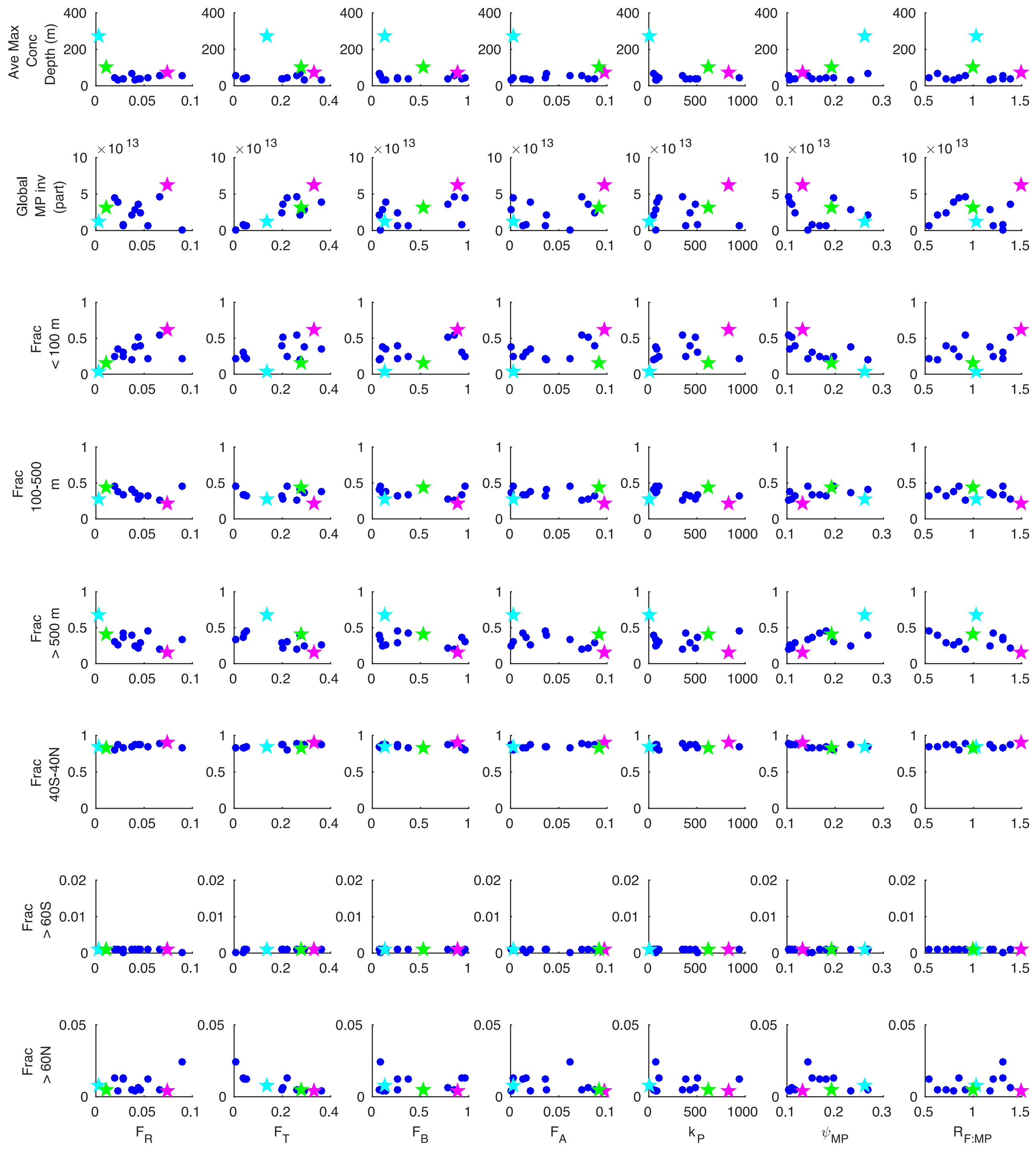

Figure S8. Fraction of the total MP inventory held within various regions as a function of MP model parameters at year 2010 after filtering the simulations based on criteria in the text. Model configurations used in the main text are shown as stars (Test100; main text TestLo:cyan, Test219; main text TestHi:magenta, Test275; main text TestMed:green).

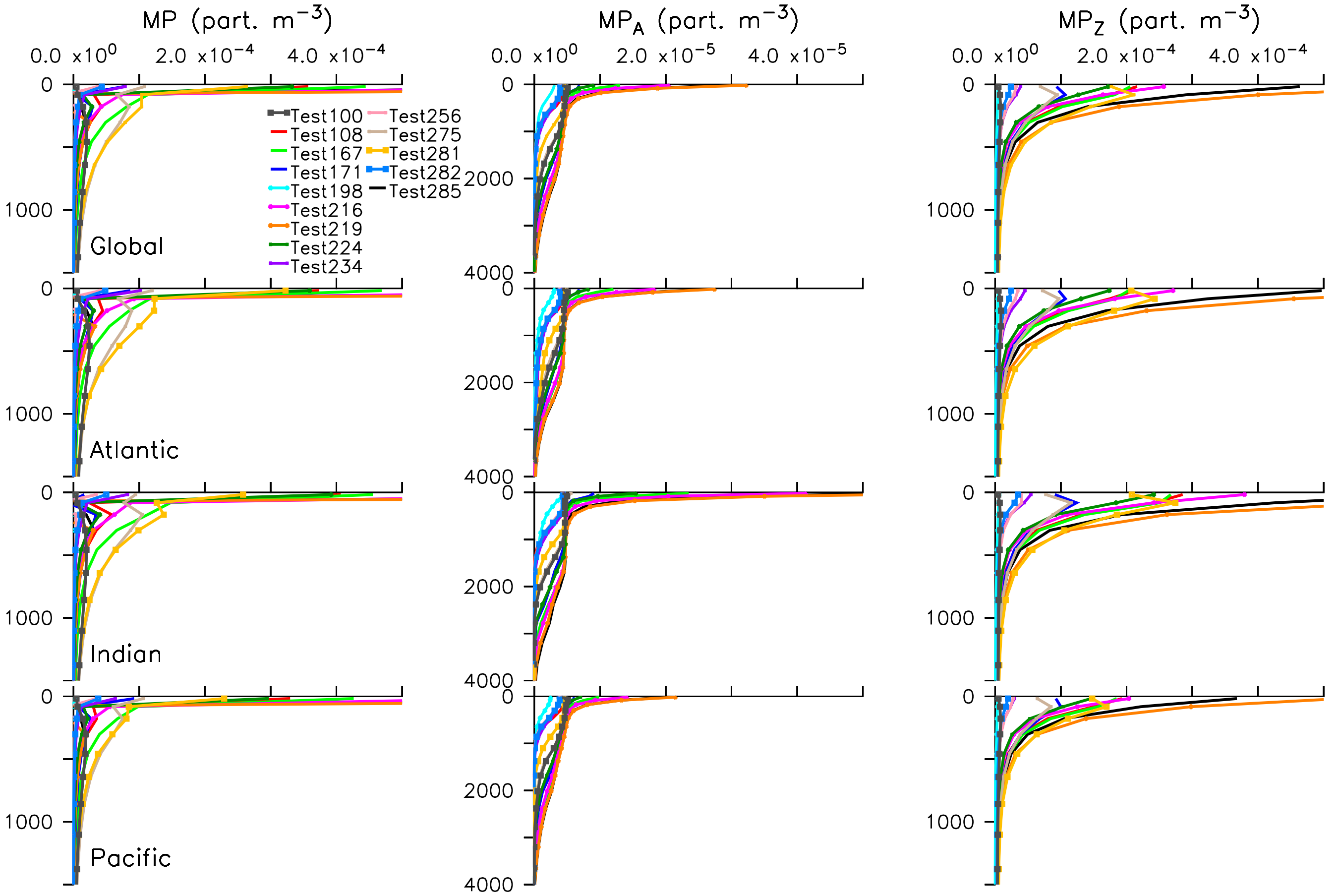

Figure S9. Average MP concentration (particles per m^3) by depth in meters for each ocean basin that contains MP at year 2010 for the filtered simulations.
